# MOF/graphene oxide based composites in smart supercapacitors: a comprehensive review on the electrochemical evaluation and material development for advanced energy storage devices

**DOI:** 10.1039/d4ra01027b

**Published:** 2024-04-30

**Authors:** Sanjeev Gautam, Shruti Rialach, Surinder Paul, Navdeep Goyal

**Affiliations:** a Advanced Functional Materials Lab, Dr S.S. Bhatnagar University Institute of Chemical Engineering & Technology, Panjab University Chandigarh-160014 India sgautam@pu.ac.in +91 97797 13212; b Department of Physics and Astronomical Science, Central University of Himachal Pradesh Dharamshala 176215 India; c Energy Research Centre, Panjab University Chandigarh-160014 India; d Department of Physics, Panjab University Chandigarh-160014 India

## Abstract

The surge in interest surrounding energy storage solutions, driven by the demand for electric vehicles and the global energy crisis, has spotlighted the effectiveness of carbon-based supercapacitors in meeting high-power requirements. Concurrently, metal–organic frameworks (MOFs) have gained attention as a template for their integration with graphene oxide (GO) in composite materials which have emerged as a promising avenue for developing high-power supercapacitors, elevating smart supercapacitor efficiency, cyclic stability, and durability, providing crucial insights for overcoming contemporary energy storage obstacles. The identified combination leverages the strengths of both materials, showcasing significant potential for advancing energy storage technologies in a sustainable and efficient manner. In this research, an in-depth review has been presented, in which properties, rationale and integration of MOF/GO composites have been critically examined. Various fabrication techniques have been thoroughly analyzed, emphasizing the specific attributes of MOFs, such as high surface area and modifiable porosity, in tandem with the conductive and stabilizing features of graphene oxide. Electrochemical characterizations and physicochemical mechanisms underlying MOF/GO composites have been examined, emphasizing their synergistic interaction, leading to superior electrical conductivity, mechanical robustness, and energy storage capacity. The article concludes by identifying future research directions, emphasizing sustainable production, material optimization, and integration strategies to address the persistent challenges in the field of energy storage. In essence, this research article aims to offer a concise and insightful resource for researchers engaged in overcoming the pressing energy storage issues of our time through the exploration of MOF/GO composites in smart supercapacitors.

## Abstract 2

Driving towards sustainable energy solutions: harnessing metal–organic framework/graphene oxide composites to propel high-efficiency smart supercapacitors.

## Graphical abstract

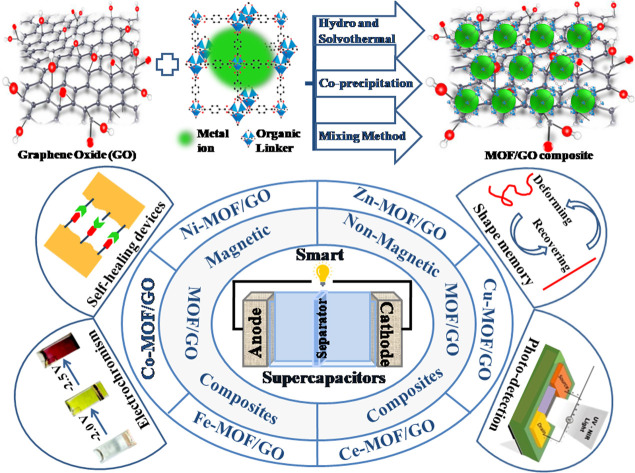

## 1 Introduction

**Fig. 1:**
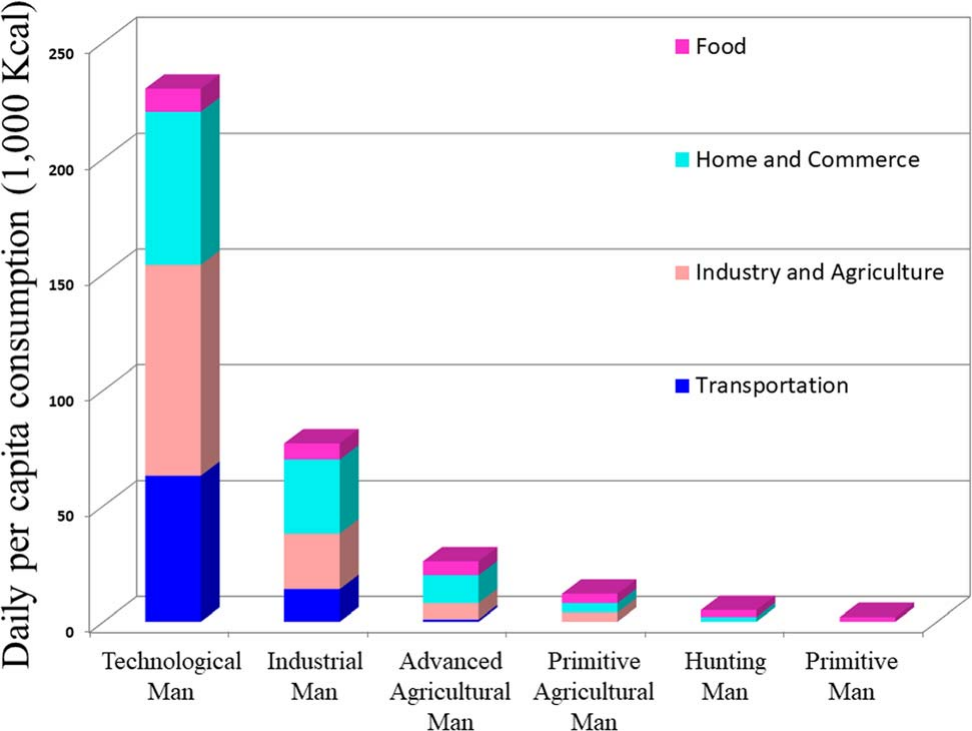
The change in energy consumption patterns over time.^1^ Adapted from the source: *Sci. Am.*, 1971, **225**, 134–147. Copyright 1971 JSTOR.

The vitality of life hinges on the acquisition and conversion of energy from the environment, a fundamental process in which human actively participate by transforming less desirable energy forms into essential resources such as meat, heat from wood, and electricity from fossil fuels. Human energy consumption evolved from primitive reliance on food to more efficient hunting and primitive agriculture. The 1875 onset of industrialization and the steam engine led to a threefold increase in energy consumption, unlocking access to concentrated solar energy storage in fossil fuels. Industrialization revolutionized energy dynamics; by 1970, daily energy consumption was 115 times higher than in prehistoric societies due to technological advancements. Fig. 1 below illustrates how patterns of energy usage have changed throughout time.

The burgeoning global demand for energy underscores its pivotal significance in enhancing societal well-being, mitigating climate change, and ensuring the requisites for sustainable human existence.^2^ The International Energy Agency (IEA) predicts a yearly increase of 3% in global energy demand throughout the period from 2023 to 2025. The question that now needs to be answered is: How are we going to sustainably meet these demands? The answer lies in energy storage! Yes, energy storage technologies allow us to store excess energy and discharge it when there is too little generation or too much demand. Energy storage plays a crucial role in ensuring a continuous and reliable supply of renewable energy to power systems, even during periods of no sunlight and low wind speeds. Growing storage systems are crucial to the global deployment of renewable energy, and when combined with variable renewable like wind and solar, they can help replace expensive and environmentally harmful fossil fuel-generated electricity while boosting supply security. In this era of rapid technological advancement and growing energy demands, the need to store energy has never been more apparent.

**Fig. 2:**
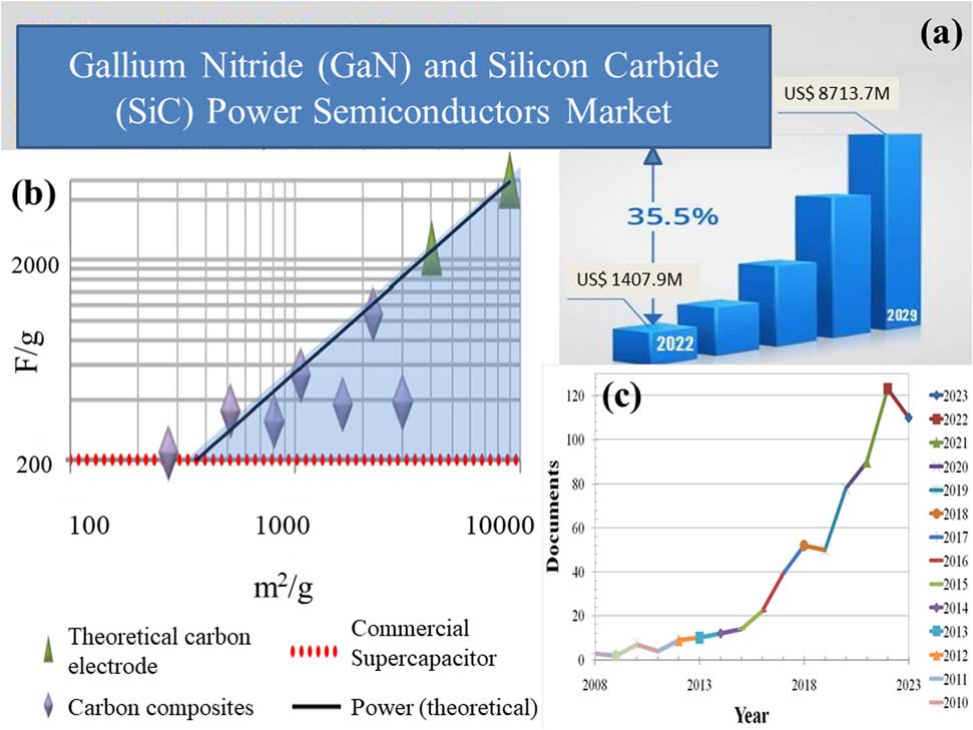
(a) The current status, forecast, and size of the global market for gallium nitride (GaN) and silicon carbide (SiC) power semiconductors,^3^ (b) recent breakthroughs in carbon allotropes as electrode materials,^4^ and (c) research articles published regarding the advancements and trends in MOF/GO composite materials for energy storage devices over the past 15 years. (a) Adapted from the source: openPR. Copyright 2022 openPR, and (b) adapted with permission from source: *Nano Energy*, 2014, **9**, 128–141. Copyright 2014 Elsevier.

Capacitors and widely available batteries constitute prominent energy storage devices within the electric market. Capacitors store energy through charge accumulation, creating a potential difference across plates. However, their energy density is constrained by dielectric limitations. In contrast, batteries store energy through reversible chemical reactions, yet face challenges with low power density during rapid cycles. Because of this restriction, scientists have been investigating supercapacitors, which provide a unique combination of outstanding power density, long cycle life, and swift charging/discharging times. These attributes render supercapacitors well-suited for various applications, *viz.*, power electronics, power amplifiers, *etc.*, necessitating expeditious energy discharges and frequent cycling. Silicon carbide (SiC) and gallium nitride (GaN) have become integral components in power electronics, as depicted in Fig. 2(a), owing to their inherent properties. The surge in demand, however, has presented supply challenges, necessitating a thorough exploration of strategies to meet the escalating needs of the rapidly expanding market. Carbon, unlike silicon and gallium, is abundant in nature. The planet's vast forests, peat bogs, and even carbon dioxide from the atmosphere can be harnessed for carbon-based materials. Fig. 2(b) illustrates recent studies on carbon allotropes employed as electrode materials. Among the most promising candidates for supercapacitor electrodes are carbon derivatives and composite materials. MOF/GO composites are gaining significant traction among researchers for energy storage applications owing to their unique combination of properties as this synergistic combination enables precise control over ion transport kinetics and electrochemical reactions, thus promising advancements in the development of high-performance energy storage devices such as supercapacitors. Fig. 2(c) provides a comprehensive overview of research articles published over the past 15 years, highlighting the advancements and trends in MOF/GO composite materials for energy storage devices.

In this paper, we have delved deeply into the dynamic world of MOF/GO composites for supercapacitor applications, pushing the boundaries of energy storage technology to new frontiers. The article commences by explaining the fabrication and charge storage mechanism in various types of supercapacitors, further examining the rationale and properties of MOFs and GO, emphasizing their individual contributions to enhancing supercapacitor performance. Notable features of MOFs, including their elevated surface area and modifiable porosity, are discussed alongside the conductive and stabilizing characteristics of graphene oxide. Subsequent sections explore fabrication techniques, energy storage mechanism and physicochemical mechanisms of MOF/GO composites, highlighting their synergistic effects on electrical conductivity, mechanical robustness, and energy storage capacity. This research stands in stark contrast to conventional energy storage devices, providing a promising trajectory for the evolution of high-performance supercapacitor technologies. Several characterization techniques frequently used to study the electrochemical behavior of supercapacitor materials and devices have also been discussed in the latter sections for comprehensive understanding of the performance and limitations of supercapacitors, facilitating the development of improved energy storage devices. Through a comprehensive discussion, this review delves into advancements that cater to the unique requirements of each application, emphasizing the evolving landscape of MOF/GO composites in smart supercapacitors. In conclusion, the review anticipates future research directions by underscoring the imperative for sustainable and scalable production methods, continued material optimization, and innovative integration strategies. These identified avenues not only hold the potential to address current challenges but also pave the way for the continual advancement of supercapacitor technologies, ensuring their relevance and efficacy in evolving energy storage landscapes.

## 2 Supercapacitors

### 2.1 Types and operation

**Fig. 3:**
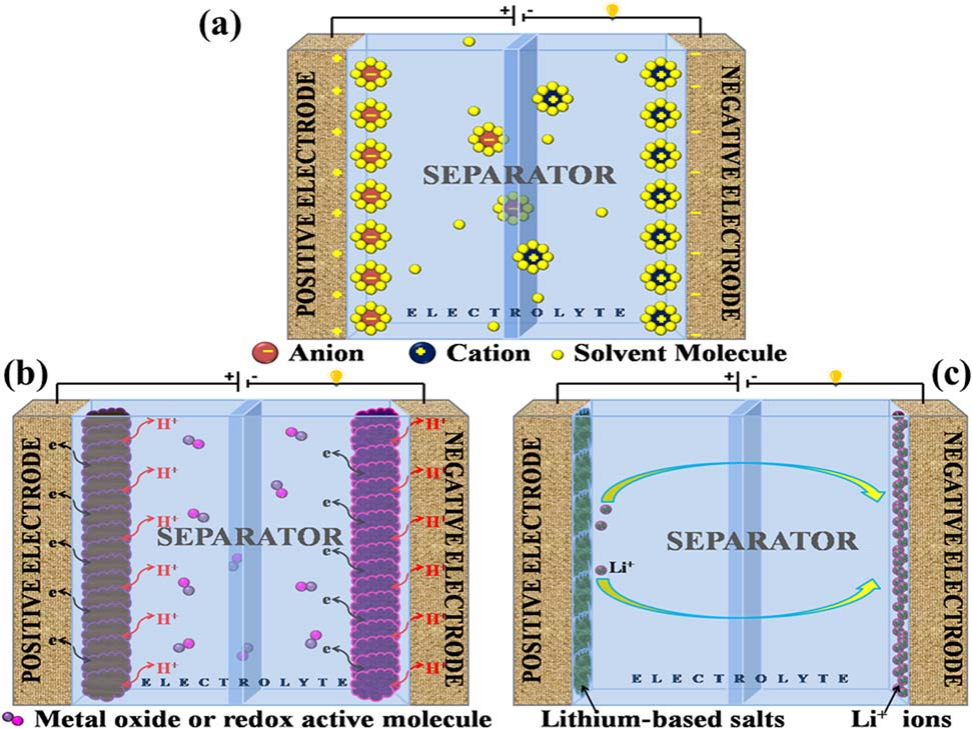
Illustration of the mechanisms for storing charge in (a) electric double-layer capacitor, (b) pseudocapacitor, and (c) hybrid capacitor.

A supercapacitor, alternatively referred to as an ultracapacitor or electrochemical capacitor, functions as an electrical energy storage device that stores energy *via* electrostatic charge separation at the interface of porous electrodes and an electrolyte. It offers a unique set of advantages, such as rapid charging and discharging abilities,^5^ an extended cycle life,^6^ and high power density.^7^ These remarkable attributes make supercapacitors a crucial component in addressing a multitude of contemporary challenges, from powering electric vehicles^8^ and renewable energy systems^9^ to enhancing the performance of portable electronics.^10,11^ A captivating recent application of supercapacitors involves ABB flash charging Swiss buses. Every time a bus smoothly comes to a stop, a contact ascends to connect with a supercapacitor flash charger, providing a rapid delivery of 600 kilowatts in just 15 seconds.^12^ In this age of innovation and environmental consciousness, the importance of supercapacitors as a key enabler of our energy-efficient, sustainable future cannot be overstated. Based on how these supercapacitors store energy, as seen in Fig. 3, supercapacitors are categorized into three distinct types.

(1) Electrochemical double-layer capacitor

(2) Pseudocapacitor

(3) Hybrid capacitor

#### 2.1.1 Electrochemical double-layer capacitor

EDLCs, known as electric double-layer capacitors, represent a category of energy storage devices that store electrical energy by electrostatically separating charges at the interface between an electrolyte and an electrode material that possesses a high surface area. Ions, both positive and negative, from the electrolyte are attracted to the surface of the opposing electrode when a voltage is applied, which is the charge accumulation mechanism in EDLCs. This division of charges creates an electrostatic double-layer at each electrode–electrolyte interface. The energy is stored as accumulated charge in the electrostatic double-layer. Ions move to the double layer's surface when voltage is applied to the electrodes, charging the capacitor. On the other hand, when a capacitor is discharged, ions are repelled. This is how the EDLC charges and discharges.^13^ EDLCs accumulate energy following the fundamental capacitor equation:

1 *Q* = *C* × *V*

In this equation, *Q* represents the stored charge, *C* denotes the capacitance, and *V* stands for the applied voltage.

The double-layer capacitance (*C*_dl_) in EDLCs is given by:2
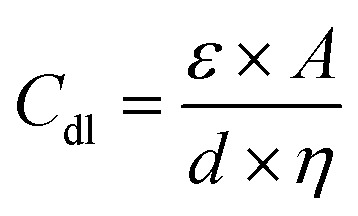


In this context, *ε* signifies the permittivity of the electrolyte, *A* corresponds to the surface area of electrodes, *d* represents the distance between electrodes, and *η* stands for the viscosity of the electrolyte.

The total capacitance (*C*_total_) of the EDLC can be calculated as the sum of inverse of the double-layer capacitance of each electrode:3
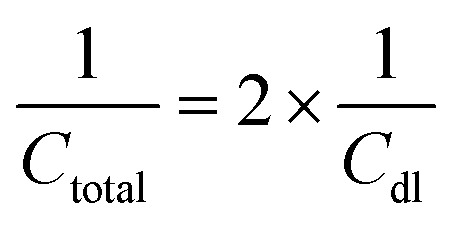
where “2” is used because there are two electrodes in an EDLC.

When a voltage (*V*) is applied across the EDLC, we can calculate the amount of charge (*Q*) it can store employing the initial equation. The energy (*E*) stored in the EDLC can be computed utilizing the subsequent equation:

4 *E* = 0.5 × *C*_total_ × *V*^2^

These equations provide a mathematical framework for understanding how charge, capacitance, voltage, and energy are related in an EDLC. The actual capacitance and energy storage capabilities depend on the specific design and materials used in the EDLC, as well as the operating conditions. The process is purely electrostatic and does not involve chemical reactions, making EDLCs capable of rapid energy storage and release, along with sustaining an enormous number of cycles of charge and discharge. However, these devices' energy density is somewhat constrained because of their charge storing technique.^14^

#### 2.1.2 Pseudocapacitor

A pseudocapacitor is an energy storage system that functions as a transitional step between a battery and a traditional electrolytic capacitor. Pseudocapacitors store charge through a combination of processes, including faradaic reactions and non-faradaic (electrostatic) mechanisms. The mathematical mechanism of charge retention in pseudocapacitors can be elucidated by considering the following components:

1. *Faradaic reactions*: pseudocapacitors often use materials with redox-active properties, such as conductive polymers or oxides of transition metals, as the components in their electrodes. Charge gets accumulated in these materials through redox (reduction–oxidation) reactions. The faradaic reactions involve the passage of electrons between the electrode and the electrolytic solution. Such reactions are typically described using the Butler–Volmer equation:

5 *I* = *k*(*C*_ox_ − *C*_red_)

In this equation, *I* represents the current (charging/discharging), *k* is the rate constant for the redox reaction, *C*_ox_ denotes the concentration of the oxidized form of the redox species across the electrode, and *C*_red_ is the concentration of the reduced state of the redox molecules across the electrode.

2. *Non-faradaic (electrostatic) mechanisms*: apart from faradaic reactions, pseudocapacitors also rely on the electrostatic storage of charge. This mechanism entails the adsorption and desorption of ions, typically cations, at the juncture where the electrode and the electrolyte interface. The mathematical description of this mechanism often relies on the Gouy-Chapman-Stern (GCS) model or the Helmholtz double-layer capacitance model.

• Gouy-Chapman-Stern Model: the GCS model considers the distribution of ions in the electrical double layer formed at the juncture contacting the electrode and the electrolyte. It uses the Poisson–Boltzmann equation to describe the capacitance and distribution of charges in the double layer.

• Helmholtz double-layer capacitance: this model simplifies the GCS model and considers a more compact double layer with a constant capacitance value.

Combining both faradaic and non-faradaic mechanisms, the overall charge storage in pseudocapacitors can be mathematically described as the sum of the contributions from faradaic reactions and the double-layer capacitance. The total capacitance (*C*_total_) of a pseudocapacitor can be expressed as:6
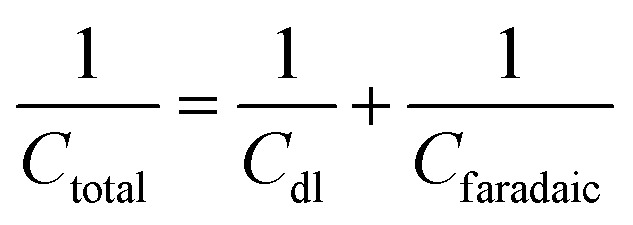


In this context, *C*_total_ represents the overall capacitance of the pseudocapacitor, *C*_dl_ is the double-layer capacitance, and *C*_f_ aradaic is the capacitance arising from faradaic redox reactions.

Nowadays, most of the pseudocapacitors store electrical energy using electrochemically active materials like conducting polymers, *viz.*, polyaniline (PANI),^15–17^ poly(3,4-propylene dioxythiophene) (PProDOT),^18,19^ poly(3,4-ethylene dioxythiophene) (PEDOT),^20–22^ polypyrrole^23–25^ and polythiophene (PT),^26–28^ and various transition metal oxides^29–31^ for fabricating the electrodes. Giving the active electrode material more specific capacitance and better energy densities is the primary benefit of the pseudocapacitor. Notwithstanding their impressive electrical characteristics, pseudocapacitors exhibit restricted electrical conductivity of the electrode material.^32^

#### 2.1.3 Hybrid capacitor

A hybrid capacitor is an assorted device for storing energy that combines the characteristics of a supercapacitor and a battery. It typically features electrodes with both electrostatic double-layer capacitance (EDLC) and pseudocapacitance properties, allowing it to offer quick charge/discharge times and a substantial amount of power density, similar to supercapacitors, while also providing higher energy density, resembling batteries.^33^ A hybrid supercapacitor's charge storage methodology integrates electrostatic double-layer capacitance (EDLC) with pseudocapacitance, which is frequently connected to redox reactions. The mathematical equations for these two mechanisms are as follows:

The EDLC component is associated with the physical separation of charges at the electrode–electrolyte juncture. The stored charge (*Q*) in an EDLC is determined by the equation:

7 *Q* = *C*_EDLC_ × *V*

where, in this equation, *Q* represents the stored charge, *C*_EDLC_ denotes the EDLC capacitance, and *V* is the applied voltage.

Pseudocapacitance results from redox processes at the electrode material's surface. The stored charge (*Q*) resulting from pseudocapacitance can be expressed using the equation:

8 *Q* = *C*_p_ × Δ*E*

In this equation, *Q* signifies the stored charge, *C*_p_ represents the pseudocapacitance, and Δ*E* is the potential difference associated with the redox reaction.

Concerning a hybrid supercapacitor, we have both EDLC and pseudocapacitance contributions. Subsequently, the combined contribution from the two mechanisms results in the total charge (*Q*_total_) contained in the hybrid supercapacitor:

9 *Q*_total_ = *Q*_EDLC_ + *Q*_p_

where, *Q*_EDLC_ is the charge stored due to EDLC, and *Q*_p_ is the charge stored due to pseudocapacitance.

The materials used in hybrid capacitors can vary depending on the specific design and application, but they typically involve the utilization of transition metal oxides and compounds based on carbon as the electrode components in view of their electrical conductivity and elevated surface area. Carbon-based materials comprise activated carbon,^34–37^ graphene,^38–41^ carbon nanotubes,^42–45^ and other carbon derivatives.^46–49^ Transition metal oxides, *viz.*, RuO_2_,^50–52^ and MnO_2_,^53–56^ can be used in the positive electrode of hybrid capacitors to increase energy storage capacity.

### 2.2 Fabrication

The fabrication of different types of supercapacitors with respect to the electrode materials, specific capacitance, cycling stability, energy density and power density values has been showcased in Table 1. In general, conducting polymers and transition metal oxides are commonly employed in the fabrication of pseudocapacitors, whereas carbon-based materials are frequently utilized for EDLC electrodes.^57^ Carbon-based EDLCs offer high power density and good cycling stability owing to their distinct working processes, yet they typically exhibit lower capacitance and energy density. Conversely, pseudocapacitors operate differently, with the electrode chemical reactions leading to the accumulation of irreversible components during cycling, thereby causing performance degradation over time. Hybrid supercapacitors (SCs) combine the charge-storage mechanisms of both EDLCs and pseudocapacitors, resulting in higher capacitance compared to EDLCs and improved cycling stability relative to pseudocapacitors. They maintain high power density while also achieving an increase in energy density.

**Table 1:** Comparison between fabrication of EDLCs, pseudocapacitors and hybrid supercapacitors

Supercapacitor	Electrode materials	Specific capacitance (F g^−1^)	Cycling stability (%)	Energy density	Power density	Applications
EDLC	Carbon-based materials (*e.g.*, activated carbon)	11.25–174	92–100	Moderate	High	Mainly in high power applications (*e.g.*, regenerative braking in vehicles)
Pseudocapacitor	MO_*x*_ conducting polymers	60–1280	67–98	Moderate to high	Moderate to high	Energy storage in portable electronics, renewable energy systems
Hybrid supercapacitor	Carbon-MO_*x*_/conducting polymers	258–2377	75–99	Higher than EDLCs typically lower than pseudocapacitors	Higher than EDLCs typically lower than pseudocapacitors	Diverse applications requiring a balance between high power and energy density

Ren *et al.* initially developed MWCNT/ordered mesoporous carbon (OMC) composite fibers as electrodes for wire-shaped supercapacitors (WSS).^58^ OMC particles grew within the interstices of MWCNTs, enhancing conductivity and enlarging the effective surface area for ion adsorption, thereby potentially increasing energy and power density. By leveraging the advantageous structures and properties of each component, the composite fiber was engineered. Subsequently, two aligned MWCNT/OMC composite fibers coated with H_3_PO_4_-PVA electrolyte were twisted to fabricate a flexible wire-shaped EDLC. Carbon microfibers (CMFs) have emerged as effective alternatives to metal wire current collectors. MWCNTs were spray-coated onto CMFs using a simple method. The resulting MWCNT/CMFs bundle served as electrodes, current collectors, and substrates or active materials for EDLCs, thus constituting a WSS. Due to the elevated effective surface area and exceptional conductivity of carbon microfibers, the resulting supercapacitors achieved heightened capacitance, power, and energy densities. Additionally, the fabrication of highly conductive films and fibers, such as super-aligned CNT (SACNT) arrays, is feasible. These materials possess versatility, serving as substrates or active materials for EDLCs, as well as highly conductive flexible current collectors.

Cui *et al.* reported the fabrication of flexible conductive textiles produced on cellulose cotton textile substrates by a straightforward “dipping and drying” method employing aqueous single-walled carbon nanotube (SWCNT) ink.^59^ Despite a high areal capacitance of 0.48 F cm^−2^, minimal capacitance loss was observed after 130 000 cycles. Traditionally, metal foils and wires have been utilized as current collectors to enhance performance; however, their weight and susceptibility to fatigue under repeated bending limit their applicability in flexible power sources. In contrast, the conductive CNT networks in this study serve as three-dimensional flexible current collectors, effectively reducing the overall bulk of the device and streamlining its construction.

MnO_2_ stands out as a top-tier pseudocapacitance material due to its exceptional properties, including a high theoretical specific capacitance of approximately 1400 F g^−1^, cost-effectiveness, low toxicity, and abundance in nature. Flexible substrates, such as sponges comprised of interconnected cellulose or polymer fibers with hierarchical macroporous characteristics, were employed for loading active ingredients by Chen *et al.*^60^ Utilizing a simple and scalable “dipping and drying” technique, carbon nanotubes (CNTs) were coated, followed by MnO_2_ electrodeposition, resulting in the fabrication of a MnO_2_/CNT/sponge electrode. The synergistic combination of the highly conductive and porous CNT-coated sponge with the porous electrodeposited MnO_2_ yielded impressive outcomes, including a higher specific capacitance of 1230 F g^−1^, a specific power density of 63 kW kg^−1^, a specific energy density of 31 kW h kg^−1^, and consistent cycling stability over 10 000 cycles with minimal capacitance decay.

Free-standing, flexible graphene films with a 3D interconnected porous structure have also been highlighted in some studies as the perfect substrates for creating hybrid SCs. Meng *et al.* generated a homogeneous mixture by incorporating CaCl_2_ into a GO dispersion. Upon bubbling CO_2_ through the mixture, CaCO_3_ particles encapsulated within GO sheets were formed.^61^ Following vacuum filtration, a GO/CaCO_3_ hybrid film was obtained. Subsequent reduction of GO using hydrazine and removal of CaCO_3_ using diluted acid resulted in the formation of a flexible 3D graphene skeletal sheet. A hierarchical 3D PANI/graphene composite film was then prepared by uniformly growing PANI nanowire arrays on both the interior and exterior surfaces of the 3D graphene substrate. Due to its interconnected porous structure, the composite film retained 88% of its initial capacitance after 5000 cycles at a current density of 5 A g^−1^. Moreover, the SC based on the composite film exhibited excellent flexibility and rate performance, highlighting the potential utility of flexible hybrid supercapacitors.

## 3 Metal–organic frameworks (MOFs)

### 3.1 Introduction to MOFs

**Fig. 4:**
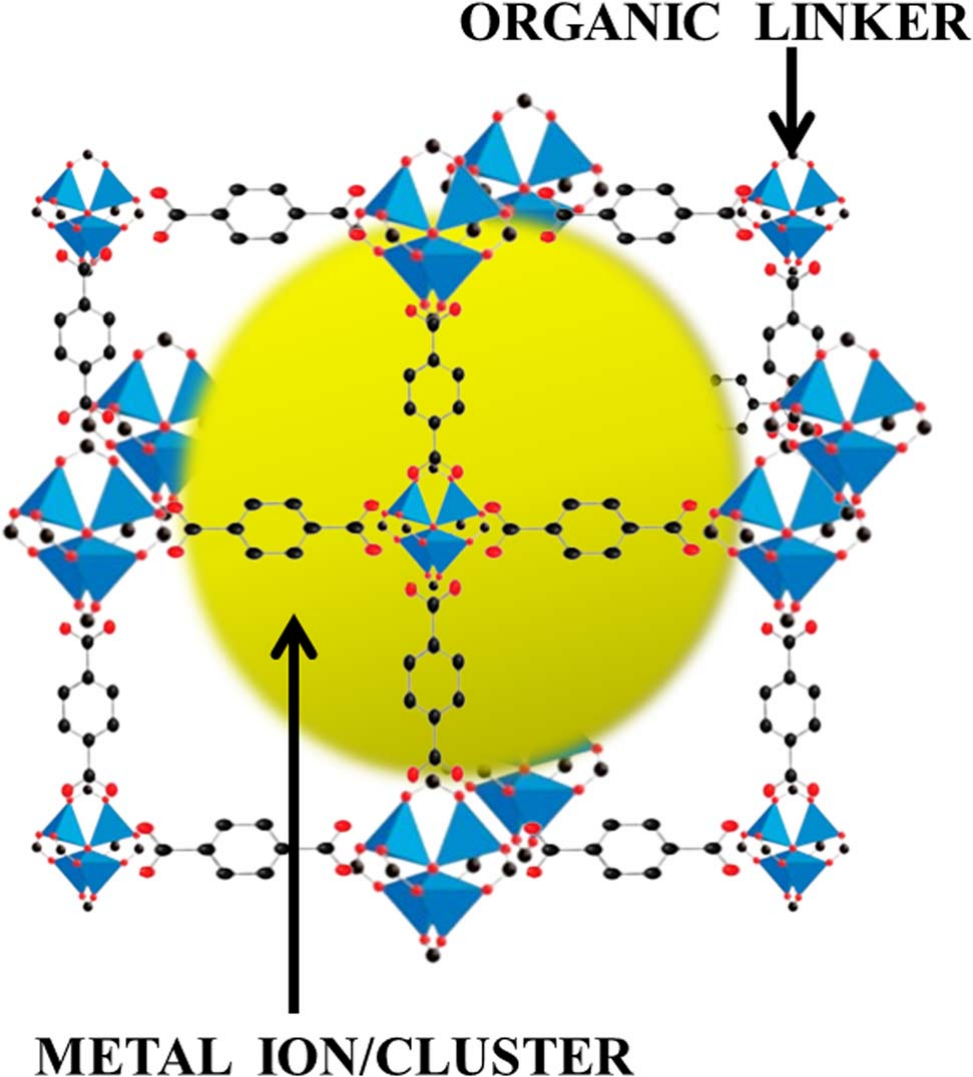
Architecture of metal–organic frameworks, weaving the metal ions/clusters and organic linkers.

Ever since the inception of highly porous MOF materials in the mid-1990s, the field of applied nanomaterials has garnered a tremendous interest in chemistry, material science, chemical engineering, and related areas.^62–68^ MOFs, as already discussed, are crystallized substances comprising metal ions or clusters which are connected to each other by organic linker molecules. They are also sometimes referred to as porous coordination polymers or coordination network solids. Here's a brief introduction to MOFs:

*Structure*: the metal nodes or clusters that are linked together by organic ligands in MOFs form a three-dimensional, periodic lattice structure. The metal nodes act as coordination centers, the organic ligands serve as connecting struts and the modular design allows for a wide variety of metals and organic linkers to be used, leading to an extensive array of MOF structures. Fig. 4 shows the MOF structure in three dimensions where the metal ion/cluster has been wrapped by the organic linker molecules.

*Porosity*: one of the most remarkable features of MOFs is their high porosity. The consistent placement of organic linkers and metal nodes creates a network of pores or cavities within the material. This porous structure is also thought of as an electrolyte membrane that permits charge transfer.^69^ These pores can be of various sizes and shapes, making MOFs suitable for a lot of applications.

*Tunability*: MOFs are highly tunable materials. Researchers can design and synthesize MOFs with specific properties by choosing numerous available metal ions and organic linkers arrangements. The prescribed tunability allows for the customization of MOFs to meet the requirements of various applications.

*Applications*: various potential applications of MOFs have been recognized across numerous industries, including drug delivery, pharmaceuticals, medical imaging, gas adsorption and separation, waste–water treatment, photocatalysis, sensors, batteries, and, lately, supercapacitors.^70–78^

### 3.2 MOFs in supercapacitors

MOFs are attracting attention in applications involving supercapacitors because of their distinctive attributes that render them well-suited for energy storage materials. Due of their substantial surface area, consistent and permanent porosity, and controllable pore dimensions and shapes, MOFs are perfect for increasing the power density and charge storage capacity of supercapacitors.^79^ Additionally, it is possible to engineer some MOFs with enhanced stability and electrical conductivity in electrolytes. This combination of features makes MOFs a material with great potential for advancing supercapacitor technology, with the potential for higher energy storage and faster charge–discharge rates. A lot of research has been done in the last several years on functionalizing pristine MOFs for use as supercapacitor electrode materials directly. For instance, Lee *et al.* developed pure metal–organic frameworks (Co-MOF) based on cobalt, which show promise as a material for supercapacitors. With a specific capacitance of up to 206.76 F g^−1^, the doctor bladed Co-MOF film demonstrated commendable pseudocapacitor performance.^80^ On the other hand, using a straightforward solvothermal technique, Kang *et al.* synthesized Ni-MOF materials created using Ni_3_(1,3,5-benzenetricarboxylic acid)_2_·12H_2_O. After 1000 cycles, it maintained a strong capacitance retention rate of 94.6% and a boosted specific capacitance of 726 F g^−1^.^81^ Despite all the features, a number of significant drawbacks have prevented MOFs from being used in practical application fields, including insufficient conductivity, inadequate chemical stability, and often high manufacturing costs.^82,83^ Making metal oxides or carbonaceous compounds from MOFs has shown promise as a solution to these pristine MOF constraints.^84^ The low conductivity intrinsic problem of pure MOFs, which hinders the achievement of appropriate charge transfer levels in supercapacitors, is resolved by these composite materials, a few of which have been discussed below and tabulated in Table 2.

**Table 2:** Different types of MOF-composites exhibiting varying power densities, energy densities, and specific capacitance values

Electrode material	Synthesis route	Energy density (W h kg^−1^)	Power density (W kg^−1^)	Cycling stability (%)/cycles	Specific capacitance (F g^−1^)	Ref.
MOF-CNT (10%)	*In situ*	20.2	750	89.5%/8000	431.6@1 A g^−1^	85
Trimetallic MOF-CNT	Solvothermal	23.6	501.5	79.2%/10 000	978.54@2 A g^−1^	86
Co-MOF/PANI	Hydrothermal	23.2	4480	100%/3000	162.5C g^−1^@2 A g^−1^	87
MOF-PEDOT	Chemical vapor polymerization (CVP)	40.6	450	80.6%/1000	1401@0.5 A g^−1^	88
Cu-MOF@δ-MnO_2_	Electrodeposition	—	—	95%/6000	340@1 A g^−1^	90
MOF-LaFeO_3_	MOF-gel method	34	900	92.2%/5000	241.3@1 A g^−1^	91
65Ni-MDH	Hydrothermal	81	1900	91.3%/10 000	875C g^−1^@1 A g^−1^	92

(a) MOF/carbon nano-tube composites: amalgamation of metal–organic frameworks (MOFs) with carbon nanotubes (CNTs) has been explored *via* several methodologies, characterized by their remarkable electrical properties, unique pore architecture, and robust mechanical and thermal properties. Very recently, a green *in situ* physical mixing technique was proposed by Zhang *et al.* to enhance the architecture of MOF/CNT (10%).^85^ Optimal composite structure depicted a notable increase in specific capacitance to 431.6 F g^−1^ at a current density of 1 A g^−1^ (based on 1 M Li_2_SO_4_ electrolyte), surpassing that of the pristine MOF (143.1 F g^−1^), anticipating that the addition of CNTs could enhance the specific capacitance and rate performance of the pristine MOF gel. Moreover, the device showcased an extended cycle life, retaining 89.5% of its capacitance after 8000 charge–discharge cycles at 10 A g^−1^, alongside a high energy density of 20.2 W h kg^−1^ at a power density of 750 W kg^−1^, suggesting that this enhancement primarily stems from the fact that an appropriate ratio of CNTs can significantly augment the electrical conductivity and ion transmission efficiency of MOFs. Anwer *et al.* also presented a trimetallic-based MOF/CNT (5%) featuring hierarchical/dual-layered structures synthesized *via* a straightforward solvothermal method.^86^ The hybrid supercapacitor demonstrated a significant power density of 501.5 W kg^−1^ and an energy density of 23.6 W h kg^−1^, which might be attributed to the synergistic features of the MOF-based composite contributing to enhanced electrochemical capabilities, including increased electro-active locations, shortened diffusion paths for electrolytes, and a higher number of redox reactions in the KOH electrolyte.

(b) MOF/conducting polymers: the intercalation of MOFs with conducting polymers (CPs) such as polyaniline (PANI), polypyrrole (Ppy), and polyethylene dioxythiophene (PEDOT) has been approached in a number of ways as the CPs have been reported to offer favorable device stability, high pseudocapacitance, and straightforward synthesis procedures. Iqbal *et al.* utilized a hydrothermal approach to synthesize a unique Co-MOF material, which was subsequently physically infused with polyaniline (PANI) to enhance the performance of the pristine material.^87^ Operating at 1 A g^−1^, parallel operation yielded a maximum specific energy of 23.11 W h kg^−1^, accompanied by a specific power of 1600 W kg^−1^ for MOF-PANI assembly. Similarly, at 4 A g^−1^, achieving a specific energy of 8.906 W h kg^−1^ corresponded to an outstanding specific power of 6400 W kg^−1^, with a specific capacitance retention of 146% after 3000 cycles which is substantiated by a theoretical Dunn's model capable of distinguishing between faradaic (diffusive) and non-faradaic (capacitive) contributions. In a different work, a highly conductive and stable MOF-PEDOT composite was successfully synthesized by Shi *et al. via* an *in situ* chemical vapor polymerization (CVP) method.^88^ The composites exhibited a very high specific capacitance of 1401 F g^−1^ at 0.5 A g^−1^, and following 1000 cycles at 10 A g^−1^, the specific capacitance of the Ni-MOF/PEDOT composites retained 80.6% of its initial capacitance. Ni-MOF contributes to a high specific capacitance, while PEDOT positively influences the structural stability of Ni-MOF, thereby significantly enhancing the cycle life of the composites. Additionally, an asymmetric supercapacitor (ASC) was constructed using activated carbon (AC) and Ni-MOF/PEDOT composite electrodes which achieved a relatively high energy density of 40.6 W h kg^−1^ at a power density of 450 W kg^−1^, which can be ascribed to the fact that PEDOT endows the composites with the ability to facilitate rapid charge transfer, thereby enhancing the dynamic response properties of ions and reducing interface resistance.

(c) MOF/graphene nanocomposites: graphene and its derivatives, including graphene oxide (GO) and reduced graphene oxide (rGO), have garnered significant attention and have largely been reported among the materials recently introduced for supercapacitors. An effective solution proposed for addressing the energy issue involves combining metal–organic frameworks (MOFs) with graphene structures.^89^ The favorable characteristics of graphene-based materials, coupled with their ease of processability and functionalization, make them highly promising candidates for integration with MOFs for various functional materials which has been discussed in detail in the sections to follow.

## 4 Metal–organic frameworks and graphene oxide (MOF/GO) composites for supercapacitors

### 4.1 Rationale and properties of MOF/GO composite materials

Energy storage has shown a great deal of interest in MOF/GO composites, especially in the advancement of supercapacitors. The rationale for developing MOF/GO composites lies in the desire to harness the advantageous properties of both MOFs and GO while mitigating their individual limitations. Table 4 shows the comparison of electrode material, electrolyte, electrochemical performances and synthesis routes employed for MOF/GO based supercapacitors. As discussed in the previous section, MOFs have exceptional surface area and tunable porosity, owing to which they can significantly increase the charge storage capacity of supercapacitors, which leads to higher energy density – a crucial parameter for energy storage devices. On the other hand, GO, as a component of the composite, boasts excellent electrical conductivity, improving the movement of electrons within the supercapacitor and facilitating efficient energy transfer.

**Table 3:** Different types of composites with graphene derivatives exhibiting varying power densities, energy densities, and specific capacitance values

Electrode material	Synthesis route	Energy density (W h kg^−1^)	Power density (W kg^−1^)	Cycling stability (%)/cycles	Specific capacitance (F g^−1^)	Ref.
rGO/MXene-PPy	Vacuum-assisted filtration	11.3	500	67.7%/10 000	408.2@10 A g^−1^	93
Chitosan/GO hydrogel	Microwave-assisted hydrothermal	31	150	98.6%/10 000	210@0.5 A g^−1^	94
GO/ZHS	Ultrasonication	158.1	236.9	73.7%/50 000	202.8@0.2 A g^−1^	95
GQDs (graphene quantum dots)	Chemical treatment	17.36	191.7	96%/3000	257@3 A g^−1^	96
3D graphene/MnO_2_ composite network	Electrodeposition	6.8	62	82%/5000	130@2 A g^−1^	97

**Table 4:** Electrochemical behaviour comparison of MOF/GO based supercapacitors

Electrode material	Electrolyte	Synthesis route	Energy density (W h kg^−1^)	Power density (W kg^−1^)	Cycling stability (%)/cycles	Specific capacitance (F g^−1^)	Ref.
Mo-MOF/GO	1 M H_2_SO_4_	Mixing	55	400	87.5%/6000	617@1 A g^−1^	101
HPNCs/rGO-80	6 M KOH	Precipitation	18	475	90%/10 000	245@1 A g^−1^	102
Fe_2_O_3_-MOF/GO	1 M H_2_SO_4_	Mixing	79.2	405	96.3%/5000	869.2@1 A g^−1^	103
Ni/Co-MOF-rGO	6 M KOH	Reflux	72.8	42.5	91.6%/6000	860@1 A g^−1^	104
Ni-BPDC/CNF	1 M H_2_SO_4_	Precipitation	48.1	1064.7	92%/5000	250.6 mA h g^−1^@1 A g^−1^	105
Ni-MOF/rGO	1 M KOH	Ultrasonication	37.8	227	59%/500	758@1.28 F cm^2^	106
Cu-MOF/G	6 M KOH	Solvothermal	34.5	1350	93.8%/1000	482@10 mV s^−1^	107
Ni-MOF/GO	6 M KOH	Solvothermal	39.43	34.29	93.5%/1000	1017@10 mV s^−1^	108
CuO_*x*_@mC@PANI@rGO	1 M H_2_SO_4_	Polymerization	—	—	70%/500	534.5@1 A g^−1^	109
Ni-MOF/rGO	6 M KOH	Hydrothermal	16.5	250	82.4%/5000	630@1 A g^−1^	99
Co-MOF/GO	6 M KOH	Precipitation	8.10	850	78.8%/1000	549.9@10 mV s^−1^	110
Zn-MOF/rGO	3 M KOH	Hydrothermal	7.1	400	87%/5000	82.5@10 mV s^−1^	111
ZIF-8/GO	6 M KOH	*In situ* growth	12.7	447	96.6%/10 000	225.0@0.5 A g^−1^	112
Ce-MOF/GO	3 M KOH + 0.2 M K_3_Fe(CN)_6_	Wet chemical	111	449.7	85%/5000	2221.2@10 mV s^−1^	113
HMRL-1/rGO	1 M Na_2_SO_4_	Ultrasonication	57.2	4380	83.3%/4000	366.6@1 A g^−1^	114

Besides integration with MOFs, GO has also been infused with various other materials for energy storage solutions, *viz.*, chitosan/graphene oxide hybrid hydrogel electrode,^94^ reduced graphene oxide/MXene-polypyrrole composite film,^93^ PPAC-graphene hydrogel electrode,^98^ and many other composites which have been listed in Table 3. Increased research endeavors are directed towards harnessing the synergistic potential of combining multiple MOFs with diverse types of graphene. This is driven by the recognition of numerous desirable structural attributes in the MOF/GO composites, including high specific surface area, well-dispersed pore sizes, and enhanced conductivity. It is widely anticipated that these innovative materials hold significant promise for applications as electrodes in the development of highly effective supercapacitors.

**Fig. 5:**
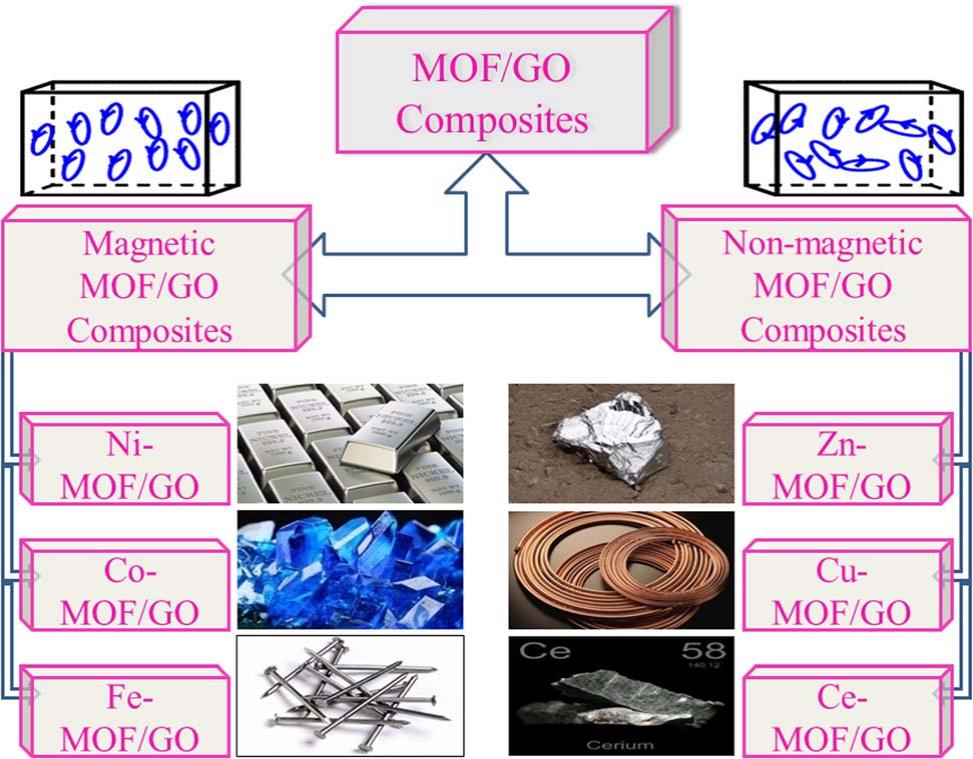
Various magnetic and non-magnetic metal MOF/GO composites used widely for supercapacitor applications in advanced energy storage materials.

We can use both magnetic and non-magnetic metals in the synthesis of MOF/GO composites depending on the specific goals and the intended use. The choice of the metal component in the MOF/GO composite will significantly impact the composite's characteristics and functionality. Fig. 5 illustrates various metal MOF/GO composites, both non-magnetic and magnetic, that can be employed in supercapacitor applications.

### 4.2 MOF/GO composites

#### 4.2.1 Magnetic metal MOF/GO composites

Magnetic MOF/GO composites incorporate magnetic metal ions or clusters into their structures. Magnetic metals impart magnetic properties to the composite, making it responsive to external magnetic fields, and can be used as the primary materials for charge transmission and storage. In addition to providing efficient ion transport channels, the thick shell of porous MOFs can guarantee the structure's mechanical durability. In the recent years, substantial amount of research has been done in this regard.

(a) Nickel-MOF/GO: in a recent work, He *et al.* synthesized Ni-BPDC/GO by employing hydrothermally produced self-made graphene oxide (*via* modified Hummer's and Offeman's method) as a substrate and 4,49-biphenyldicarboxylic acid (BPDC) to serve as an organic binder.^99^ There was no effect of GO on the growth and crystalline arrangement of Ni-BPDC (NiB). Various samples with different GO concentrations were synthesized and the calculations show that at 1 A g^−1^, the specific capacitances of NiB, NiB/GO-1, NiB/GO-3, and NiB/GO-5 are, respectively, 460 F g^−1^, 265 F g^−1^, 630 F g^−1^, and 228 F g^−1^. It is evident that NiB/GO-3 exhibits a larger portion of capacitance compared to the other two (NiB/GO-1 and NiB/GO-5), primarily because of the enhanced uniformity and even distribution achieved through its composite structure of BPDC and GO. The cyclic stability of the three-electrode system was found to be 56.5% within the first 2400 cycles and thereafter it had a constant stability of 100% after 10 000 cycles. The NiB/GO-3 composites showed high energy density value going up to 16.5 W h kg^−1^ with respect to a power density value of 250 W kg^−1^, which must be attributed to the inclusion of GO in the sample which offered abundant active sites for its functionalization with MOF molecules, which improved the specific capacitance, operational current density and rate capability in MOFs by reducing intrinsic resistance, ion diffusion impedance, and charge-transfer resistance, indicating that NiB/GO-3 composites are an appealing material for electrodes in supercapacitor applications.

Following the development of Ni-BPDC/GO, Ibrahim's group reported the development of a hierarchical structure of Ni-MOF/GO encompassing graphene nanoplatelet (GNP) adopting a mix of microwave-assisted synthesis and *in situ* approach.^100^ Initial research looked at the Ni-MOF/GO/GNP nanocomposites with varying GO amounts (Ni-M/GO_*x*_/GNP) as an electrode material for supercapacitors; Ni-M/GO_3_/GNP performed the best. Next, using a current density of 1 A g^−1^, the authors built Ni-M/GO_3_/GNP coin cell and pouch cell supercapacitors with specific capacitance values of 102.24 F g^−1^ and 70.41 F g^−1^, respectively, because GO provided a large surface area that made the electrolyte ions highly accessible. Notably, compared to the pouch cell configuration, the coin cell configuration had a 1.5-fold longer discharge time and a 50-fold greater surface under the CV curve. This is ascribed by the fact that pouch cells are put together using the polyethylene bags which leads to unwanted volume expansion throughout the charge–discharge process which negatively impacts the supercapacitor's performance. Both the cells exhibited remarkable capacitance retentions of 85.6% and 82.5%, respectively, after more than 20 000 cycles, indicating that the substrate matrices of Ni-MOF remained stable throughout the electrochemical procedures. In the coin cell assembly, the addition of GNP as well as GO has demonstrated an improvement in discharging time of approximately six and three times greater than that of the pristine Ni-MOF electrode, respectively. The improved conductivity and compositional durability of Ni-MOF might be largely accredited to the peculiar synergistic interaction that exists among the two different graphitic carbon networks included in the Ni-M/GO_3_/GNP composite. Additionally, at 993.54 W kg^−1^ power density and a noticeably larger energy density of 14.02 W h kg^−1^, the samples demonstrated why they are an ideal electrode material to feed coin cell and pouch cell systems.

(b) Cobalt-MOF/GO: cobalt based MOF/GO composites have also shown promising results as supercapacitor electrodes. Azadfalah *et al.* created a novel Co-MOF/G, using the metal linker 2-methylimidazole (2-MeIm) employing a straightforward one-step precipitation process, named as CoM/G.^110^ Owing to the integration of CoM and graphene, the electrochemical tests suggested that the available surface area, electrical conductivity, and area under the CV curves of CoM/G were significantly higher compared to those of CoM. When employed as the material for a supercapacitor electrode, an excellent specific capacitance (*C*_s_) of 549.96 F g^−1^ was recorded in a three-electrode system when the cyclic voltammetry test scan rate was 10 mV s^−1^. This value was notably larger than the observed specific capacitance (*C*_s_) of 260.75 F g^−1^ for CoM due to the addition of graphene to it which made more active sites available thus causing an increase in the nanocomposite's specific capacitance by enhancing the diffusion and proximity amongst the electroactive materials and electrolyte ions. High power density value of 850 W kg^−1^ and energy density value of 8.10 W h kg^−1^ were also demonstrated by CoM/G which is ascribed by the synergistic action of CoM and graphene in the nanocomposite. CoM/G also demonstrated an excellent cycle lifespan; that is, 78.85% of its original specific capacitance remained unchanged after 1000 cycles of charging and discharging at 1 A g^−1^, which is due to the composite structure's mechanical stability and the type of faradaic charge storage it possesses.

Chen's team has recently introduced an innovative method for developing graphene oxide-based cobalt–metal organic framework (Co-BTC@GO) aimed at enhancing the supercapacitor performance.^115^ This approach is initiated through the utilization of GO made using improved Hummer's method. Different GO dosages were injected into Co-BTC and the best results were acquired at GO dosage of 0.02 g (Co-BTC@GO:2). Demonstrating excellent rate capability, the highest specific capacitance reached 1144 F g^−1^ at 1 A g^−1^, marking a notable increase compared to the specific capacitance of 759.2 F g^−1^ observed in the sample without GO, ascribed by the superior electron transport resistance, ion diffusion rate, and stereoscopic structure of the Co-BTC@GO:2 composite as compared to Co-BTC. Moreover, Co-BTC@GO:2 demonstrated an exceptional cycle performance after 2000 cycles, keeping 88.1% of its baseline capacity after 2000 cycles, compared to 78.5% for Co-BTC, showing superior electron and charge transfer capabilities due to the fusion of three-dimensional architecture of Co-BTC microspheres with the two-dimensional lamellar membrane architecture of GO. This material is expected to provide insights into the development of electrochemically sound supercapacitor electrodes.

(c) Iron-MOF/GO: Xu's group, in 2017, came up with a straightforward mixing procedure to create a generic and easy way to make different three-dimensional GO/MOF composites on a large scale.^103^ Orange coloured Fe-MOF crystals were prepared using a simple mixing method. Thereafter, to create a Fe-MOF/GO composite hydrogel, in brief, the produced 20 mg of iron-MOF crystals were promptly added to an aqueous GO dispersion while being vigorously shaken with a vortex mixer. Following that, a freeze-dry procedure was applied to the generated composite hydrogel in order to expel the water while producing the composite aerogel (FeM/GO). At 1 A g^−1^, the FeM/GO composite electrode produced an elevated specific capacitance of 869.2 F g^−1^, while the electrode composed solely of Fe_2_O_3_ produced a significantly lower *C*_s_ of 258.5 F g^−1^ at 1 A g^−1^, which is due to a substantial electric double-layer capacitance provided by the networks made of connected GO sheets. High power densities of 8010 W kg^−1^ and 79.2 W h kg^−1^, respectively, were obtained by the FeM/GO electrode at energy densities of 25.8 W h kg^−1^ and 405 W kg^−1^, which is explained by the addition of GO, which improves the electrical conductivity and three-dimensional structure of the FeM/GO composite. The composite electrode's electrochemical stability was also evaluated by continuous charging/discharging tests at 20 A g^−1^ spanning 5000 cycles. The composite electrode had good cycling performance in the subsequent cycles, with no noticeable shift in specific capacitance after a modest increase during the first 500 cycles, which is ascribed by the significant concentration of active surface area of the nanoparticles which provides faradaic capacitance to the composite.

#### 4.2.2 Non-magnetic metal MOF/GO composites

Non-magnetic MOF/GO composites do not contain magnetic metal ions but instead use non-magnetic metals or metal clusters (*e.g.*, Zn, Cu, Pb, Al) and do not contribute magnetic properties to the composite.

(a) Zinc-MOF/GO: for a supercapacitor to be commercialized, the electrode material needs to possess a superior structure, be relatively straightforward to synthesize, be abundantly available, and exhibit outstanding performance. Thi *et al.* synthesized Zn-MOF/rGO hybrid composites produced using a hydrothermal process that have visually appealing wrinkled nanosheet-like architectural electrodes.^111^ Zinc-MOF was synthesized using *p*-benzenedicarboxylic acid (H_2_BDC), and two types of composites – Zn-BDC/rGO10 and Zn-BDC/rGO20 – were made depending on the amount of rGO. With a three-electrode configuration, the electrochemical capacitive efficiency was evaluated in a 3 M aqueous KOH electrolyte. At 1 A g^−1^, Zn-BDC/rGO20 had a greater specific capacity than Zn-BDC/rGO10 and pure Zn-BDC, with respective values of 205, 153, and 54C g^−1^, and it is evident that the incorporation of rGO raised the electrodes' specific capacitance. Specifically, Zn-BDC/rGO20 had a specific capacity that was almost twice as high as pure Zn-BDC. In addition, the Zn-BDC/rGO20 symmetric device showed a specific capacitance value of 82.5 F g^−1^ at a power density level of 0.4 kW kg^−1^ and an energy density level of 7.1 W h kg^−1^ owing to the engagement of metal centers in graphene nanosheet-mediated fast electron transfer processes. Zn-BDC/rGO composites exhibit excellent electrochemical performance and can be easily synthesized, making them promising materials for supercapacitor electrodes.

In a different report, Wang *et al.* developed Zn-MOF/GO using 2-methylimidazole as an organic linker.^112^ Self-made GO by modified Hummers' method was mixed with Zn-MOF and the solution was named ZIF-8/GO. The sample yielded an enormous surface area of 816.4 m^2^ g^−1^ owing to the large accessible electrolytic surface provided by ZIF-8 nanoparticles. High specific capacitance levels of 225.0 F g^−1^ at 0.5 A g^−1^, improved rate capabilities, and outstanding electrochemical reliability exhibiting 96.8% preservation when subjected to 10 A g^−1^ were all displayed by the electrode material. This is attributed to the presence of negatively charged GO sheets which have a lot of functional groups that contain oxygen which interact with positively charged ZIF-8 nuclei to support the ZIF-8 structure anchoring on GO sheets electrostatically. An energy density value of 12.7 W h kg^−1^ with a specific power of 447 W kg^−1^ was provided by the symmetry supercapacitor. The energy density measurements maintained 6.5 W h kg^−1^ even when the power density levels rose to 15 126 W kg^−1^ as the ZIF-8 nuclei effectively prevented the restacking of GO sheets. Besides this, after 10 000 cycles, the symmetry supercapacitor demonstrated outstanding cyclic stability, retaining 96.6% of its initial capacitance at 2 A g^−1^, indicating excellent electrochemical behaviour using GO as substrate.

(b) Copper-MOF/GO: Singh *et al.*, in 2021, synthesized a new biporous blue coloured crystals of Cu-MOF by solvothermal route whose composite with GO was made using simple ultrasonication technique.^114^ The composite exhibited good measurements of specific capacitance value of 366.6 F g^−1^ and showed a dual capacitive behavior at 1 A g^−1^ current density range, falling to 83.30% spanning 4000 cycles, displaying the improved electrochemical properties brought about by the combination of rGO and MOF, without compromising thermal or mechanical stability. In view of achieving better charge storage performance, the symmetrical supercapacitor device demonstrated an optimal power density of 21.10 kW kg^−1^ at 14.66 W h kg^−1^ and an optimal energy density value of 57.2 W h kg^−1^ at 4.38 kW kg^−1^, ascribed by the permeable MOF's redox-active Cu^2+^ centers, which have appropriate channel architectures to aid in ion transport. The observed outcomes suggest that the freshly developed Cu-MOF/GO might be a promising component that can be composited with other conducting matrices.

Recently, Sarathkumar's group prepared nitrogen-rich Cu-MOFs decorated on reduced GO nanosheets (Cu-MOF/rGO).^116^ The 3D porous Cu-MOFs were synthesized by facile ultrasonication over the rGO surface. The developed binder-free electrode had an excellent specific capacitance value of 867.09 F g^−1^ at 1 A g^−1^, which is attributed to the narrow microporous distribution in addition to a multimodal mesoporous distribution of Cu-MOF. Given that they lower the material's internal impedance and promote ion transport, these meso- and micropores are crucial textural features that improve the material's supercapacitive performance. Given that they lower the material's internal impedance and promote ion transport, these meso- and micropores are crucial textural features that improve the material's supercapacitive performance. After 5000 charging–discharging cycles, the device was observed to have a capacity retention of 131.65%, confirming its outstanding cycle stability as a consequence of the frequent cycling that activates it electrochemically. Moreover, the constructed symmetric supercapacitor device exhibited high energy density measurements of 30.56 W h kg^−1^ and power density measurements of 12 kW kg^−1^ because of the presence of Cu(ii) centers, which enables GO's redox activity to produce high energy density in addition to providing a high surface area for improved ion adsorption. The composite's exceptional electrochemical properties validate its viability for use in energy storage.

(c) Cerium-MOF/GO: Ramachandran *et al.* developed the large mesoporous structured Ce-MOF/GO composites with little alteration of literature,^117^ with Ce-MOF forming a uniform sized nanorod structure.^113^ The SEM analyses revealed that the Ce-MOF nanorods had been utilized to embellish the wrinkled GO sheets. For the prepared composite, an optimal specific capacitance value of 2221.2 F g^−1^ and an energy density value of 111.05 W h kg^−1^ were achieved in 3 M KOH + 0.2 M K_3_Fe(CN)_6_ electrolyte for a current density range of 1 A g^−1^, owing to the incorporation of redox additives. The extensive mesoporous framework of Ce-MOF and the pseudocapacitance that comes from GO sheet are responsible for this increased electrochemical performance. Ce-MOF/GO composite demonstrated exceptional long-term stability, indicating that it is an intriguing material for electrodes to feed applications involving supercapacitors.

## 5 Synthesis of integrated MOF/GO composites

**Fig. 6:**
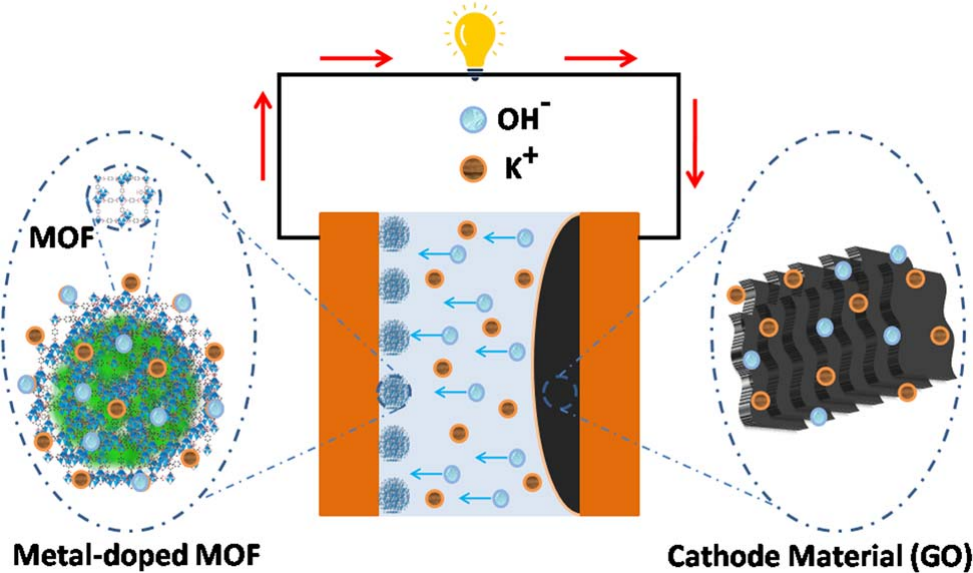
Energy storage mechanism of a supercapacitor based on MOF/GO composites.

MOFs are obtained from metallic clusters linked by organic ligands, while GO is derived from graphite and consists of oxygen-functionalized carbon sheets. The synthesis of MOF/GO composites typically involves integrating MOF crystals onto the surface of GO sheets, enhancing their capabilities. Fig. 6 shows the energy storage mechanism of a supercapacitor based on MOF/GO composites which can be made using a variety of techniques, each with its own benefits and drawbacks. These include post-synthesis,^118–121^*in situ* growth,^122–126^ solvothermal,^127–131^ co-precipitation,^132,133^ mixing,^134,135^ and ultrasonication.^136–140^

Supercapacitor applications require careful consideration of various factors in order to make certain that the finished product possesses the appropriate electrochemical properties and performance. Quite a few methods for composite synthesis are suitable for this specific application due to material compatibility, structural control, scalability and electrochemical performance. The methods that are used commonly have been listed below because they offer better control over the composite's properties and are more suited for supercapacitor applications. Besides these conventional methods, some unconventional approaches have also been discussed alongside.

### 5.1 Hydrothermal and solvothermal methods

Hydrothermal and solvothermal methods are two of the most commonly used methods for MOF/GO composite synthesis. Hydrothermal method is a synthesis technique that uses high-temperature and high-pressure water as a reaction medium to create or modify materials. Whereas, water is substituted with organic solvents in the solvothermal technique to conduct high-temperature and high-pressure reactions for material synthesis or modification.

**Fig. 7:**
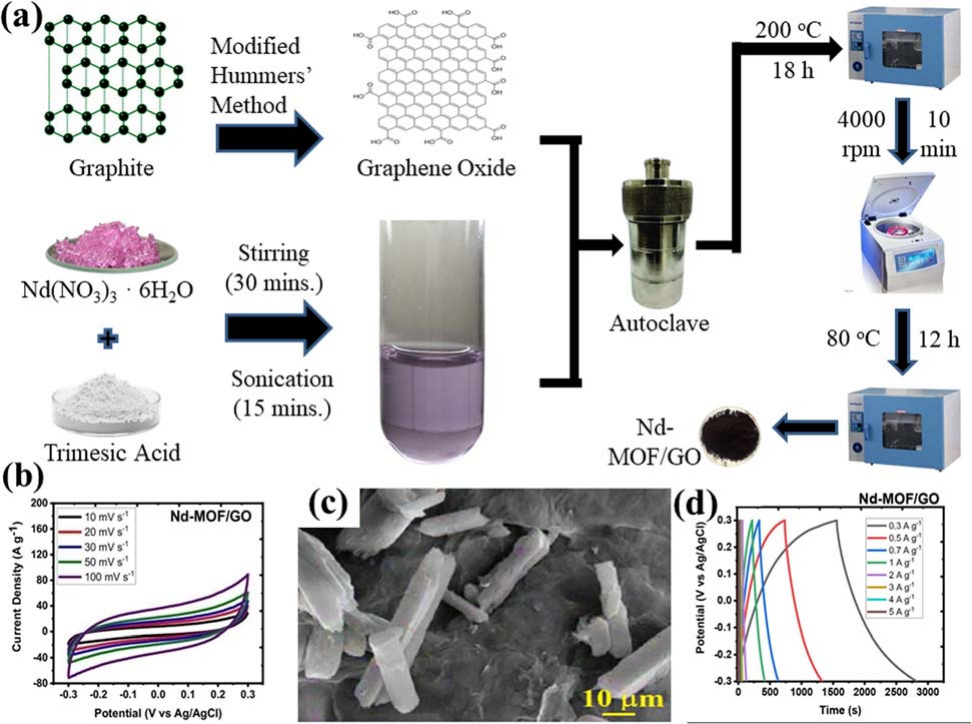
(a) Schematic presentation of synthesis of Nd-MOF/GO composites *via* hydrothermal method, (b) cyclic voltammetry of Nd-MOF/GO, (c) scanning electron microscopy micrograph of Nd-MOF/GO, and (d) galvanostatic charge/discharge of Nd-MOF/GO.^141^ (b)–(d) Adapted with permission from source: *Mater. Sci. Eng., B*, 2023, **295**, 116530. Copyright 2023 Elsevier.

Recently, Siddiqui *et al.* reported the development of rod-shaped neodymium-MOF/GO composites using simple and economical hydrothermal technique.^141^ Firstly, GO was made by them using the modified Hummers' method. The final mixture of Nd-MOF precursors and GO solution was poured into an autoclave lined with Teflon and baked for 18 hours at 200 °C celsius. Precipitates with a grey hue were produced as the autoclave cooled to ambient temperature. To maintain a pH of about 7, the precipitates were thoroughly cleaned several times and were centrifuged at 4000 rpm for about 10 minutes using a solution of ethanol and DI water. After washing, the precipitates were put in an oven and aged in a Petri dish for 12 hours at 80 °C to obtain Nd-MOF/GO, as the illustration in Fig. 7(a) shows. The Nd-MOF/GO composite's SEM picture, displayed in Fig. 7(c), features rod-like structures with a diameter of roughly 10 μm and a length of 60 μm. The electrode made up of these composites demonstrated a cyclic stability of 88.67% spanning 4000 cycles and a coulombic efficiency of 95.1%, presenting it as an appealing active material to feed supercapacitor electrodes. It also had a specific capacitance level of 633.5 F g^−1^ during a current density value of 0.3 A g^−1^, which was quite enormous. The superior capacitive response can be ascribed to the noble interconnections formed by the Nd-MOFs/GO composite, which improve its potential in a pseudo-capacitive response by enhancing the poor conductivity of the needle-like structures of Nd-MOFs. This mainly initiates the charge transport process, which leads to a stable dynamics and steady method for handling ions in the electrolyte, hence lowering diffusion resistance.

**Fig. 8:**
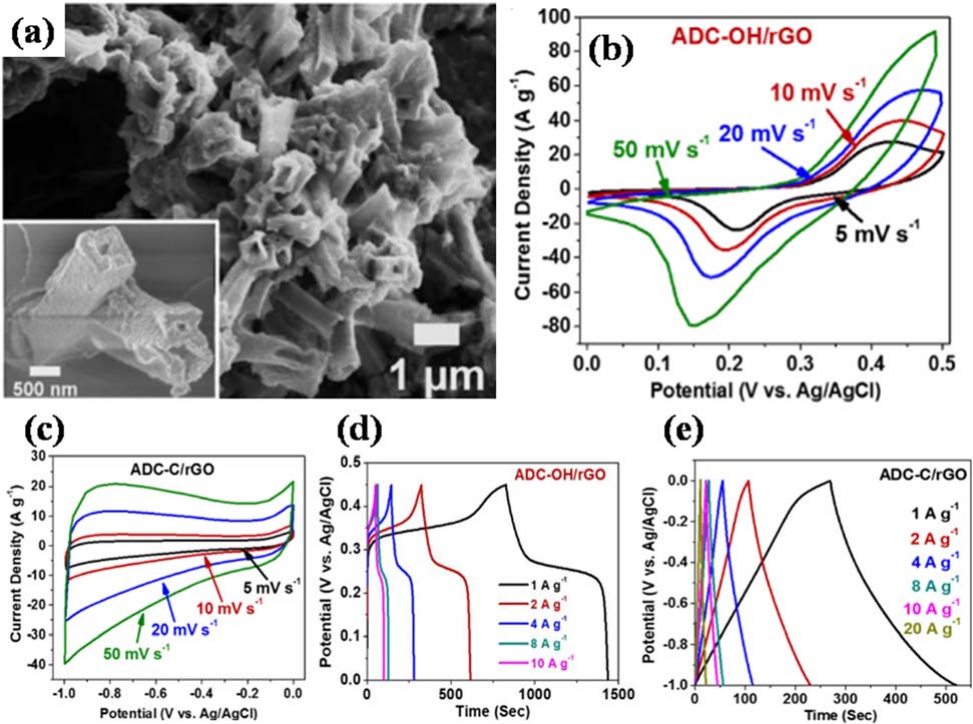
(a) Scanning electron microscopy micrograph of NiM-OH/rGO, (b) cyclic voltammetry curves of NiM-OH/rGO at different scan rates in 2 M KOH, (c) cyclic voltammetry curves of NiM-C/rGO at different scan rates in 2 M KOH, (d) galvanostatic charge/discharge curves of NiM-OH/rGO, and (e) galvanostatic charge/discharge curves of NiM-C/rGO spanning various current densities.^142^ (a)–(e) Adapted with permission from source: *ACS Appl. Energy Mater.*, 2019, **2**, 5029–5038. Copyright 2023 American Chemical Society.

In a different work reported by Jiao's group, by using a one-pot solvothermal procedure, nickel-based pillared MOFs containing rGO (NiM-rGO) composites were created.^142^ Using MOF-rGO as the precursor, various treatments were applied to produce both positive (NiM-OH/rGO) and negative (NiM-C/rGO) electrodes. Upon alkaline treatment, solid microrods in OH/rGO underwent a structural transition into hollow microrods, as demonstrated by SEM analysis (Fig. 8(a)). The resulting hybrid supercapacitor demonstrated high energy value of 59 W h kg^−1^ and power density value of 15.5 kW kg^−1^, showing a positive connection with rising scan rates, owing to the device's good rate capability. Additionally, it demonstrated remarkable stability during cycling with 95% capacity preservation spanning 10 000 cycles at 20 A g^−1^ and a decent rate ability (19% capacity loss ranging 1 to 20 A g^−1^), which is ascribed to the integration of conducting graphene sheets and seeded N species, offering an efficient electrode material. Fig. 8(b)–(e) displays the GCD and CV plots for the positive (NiM-OH/rGO) and negative (NiM-C/rGO) electrodes. The results from this study indicated that the addition of rGO to pillared MOFs markedly improved the electrochemical functionality of the derived electrode materials.

**Fig. 9:**
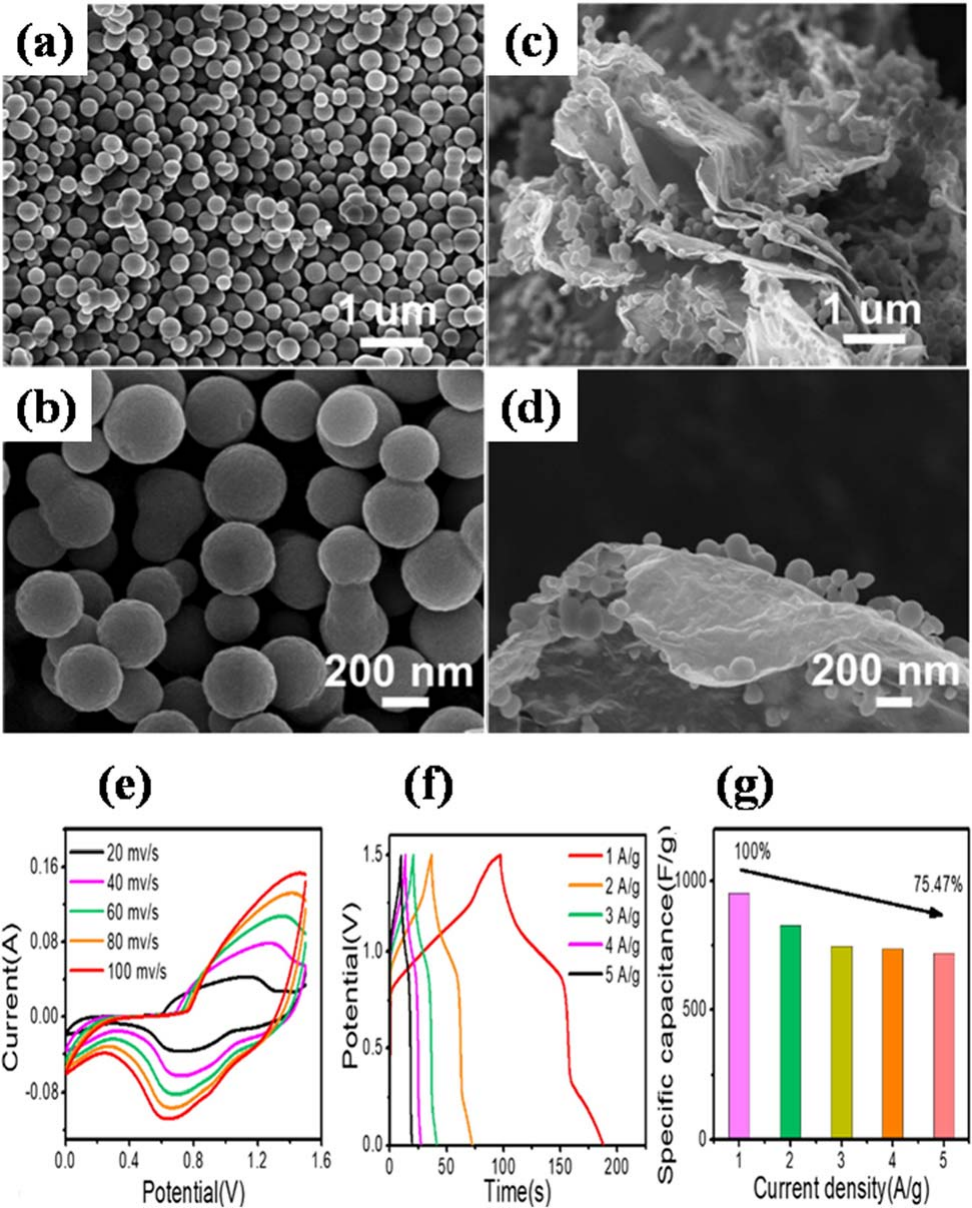
(a) and (b) Scanning electron microscopy images of monodisperse and uniform Ni-MOF nanospheres at different magnifications and angles, (c) and (d) scanning electron microscopy images of porous Ni-M/rGO300 nanospheres at different magnifications and angles, (e) the asymmetric supercapacitor's cyclic voltage measurement curves spanning various scan rates, (f) galvanostatic charge/discharge curves of the asymmetric supercapacitor spanning various current densities, and (g) obtained specific capacitance values of the asymmetric supercapacitor.^143^ (a)–(f) Adapted with permission from source: *J. Colloid Interface Sci.*, 2022, **286**, 116032. Copyright 2022 Elsevier.

Zhong *et al.* also utilized a straightforward hydrothermal and sequential calcining route to design and produce nickel-MOF microspheres that are firmly anchored upon rGO.^143^ Using a modified Hummers' technique, they used pristine graphite powder to create GO. The hydrothermal route was employed to create the Ni-MOF composite. A dark suspension was typically produced by mixing certain quantities of dried GO, Ni(NO_3_)_2_·6H_2_O, and PTA in DMF and ethylene glycol solution while stirring continuously for three days. Then the final product was enclosed within a Teflon-lined 50 mL autoclave and subjected to a hydrothermal reaction for six hours at 120 °C. Eventually, centrifuging was used to produce the black precipitate, which was then recovered after being repeatedly washed with DMF and then further dried at 60 °C. The resulting black precipitate was further calcined for one hour at 200 °C, 300 °C, and 400 °C in a pure nitrogen environment, ramping at a rate of 1 °C per minute. These products were designated as Ni-M/rGO200, Ni-M/rGO300, and Ni-M/rGO400, respectively. The morphologies of the acquired samples were analysed by SEM. The Ni-MOF spheres were uniform and monodisperse, with a typical diameter of particles of about 300 nm, as seen in Fig. 9(a) and (b). The as-formed Ni-M/rGO300 (Fig. 9(c) and (d)) displayed an increasingly permeable morphology after annealing, whereas rGO displayed a more compact structure of layers. Ni-M/rGO300 showed more folds with rGO and a relatively more consistent arrangement of Ni-MOF when compared to other morphologies. An asymmetric supercapacitor (ASC) was constructed with the Ni-M/rGO300 composite as the cathode, the anode was activated carbon (AC), and the electrolyte was KOH aqueous solution (6 mM) in order to assess the possible applications of the prepared Ni-M/rGO300 composite. Fig. 9(e) displays the CVs of the ASC at various scan speeds ranging from 20 mV s^−1^ to 100 mV s^−1^, covering a wide potential window of 0 to 1.5 V. The operating window of ASC gadget is three times wider than that of the Ni-M/rGO300 electrode in a configuration of three-electrode system, and it is evident that the CV curves of the device maintain their original forms as scan rates increase. Relatively fewer devices are needed to reach the requisite high voltage window thanks to the high voltage. The completed Ni-M/rGO300/AC ASC device's galvanostatic charge–discharge plots at varied currents of 1 to 2 A g^−1^ have been displayed in Fig. 9(f). All of the GCD curves' identical forms reflect how well the storage of charge is balanced. Additionally, the specific capacitance level of the ASC was established using the discharging times indicated in the GCD curves. The completed ASC, as depicted in Fig. 9(g), demonstrated a remarkable specific capacitance of 954 F g^−1^ at an initial current density level of 1 A g^−1^ and sustained at 720 F g^−1^ at 5 A g^−1^, demonstrating a good rate capability of 75.47%. This research envisions a wide range of opportunities for the creation of novel electrode materials that satisfy strict requirements for power density and energy content needed for devices to store energy.

### 5.2 Co-precipitation method

Co-precipitation is a method in which two or more precursor chemicals are dissolved in a solvent, and a precipitating reagent is introduced to induce the simultaneous formation of solid particles containing both components. In the context of MOF/GO composite preparation, co-precipitation process involves the precipitation of both the metal ions (for MOF) and graphene oxide in a single step. This method is preferred to hydrothermal synthesis because it is simpler, cost-effective, results in better dispersion of graphene oxide within the MOF matrix, and offers better authority over the architecture and chemistry of the resulting MOF/GO composite.

**Fig. 10:**
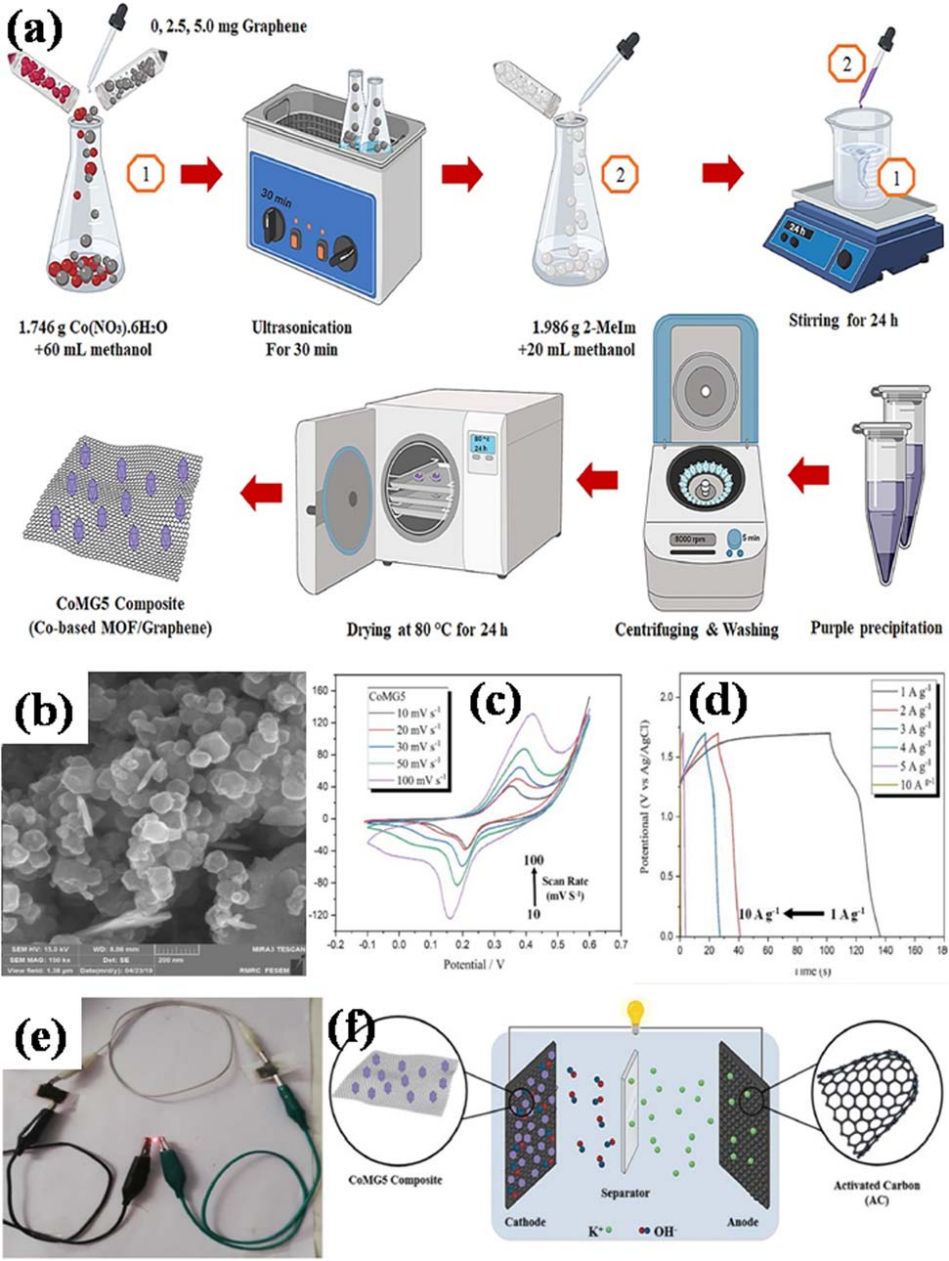
(a) Diagram illustrating the Co-M/GX nanocomposites' manufacturing process, (b) Co-M/G5 nanocomposite's micrograph obtained using field effect scanning electron microscopy, (c) the Co-M/G5 electrodes' cyclic voltammetry measurements in 6 M KOH at various scan rates (10–100 mV s^−1^) (d) plots illustrating the cell's galvanostatic charging and discharging at a current density of 1 A g^−1^, (e) the red LED illuminated by the Co-M/G5//CA device, and (f) electrode design of the Co-M/G5//CA asymmetric supercapacitor.^110^ (a)–(f) Adapted with permission from source: *J. Energy Storage*, 2021, **33**, 101925. Copyright 2021 Elsevier.

Azadfalah *et al.* employed a one-step basic precipitation route to synthesize nanocomposites of Co-based MOF/graphene.^110^ Co(NO_3_)_2_·6H_2_O was introduced into methanol, and the mixture was agitated for 15 minutes. The synthesis media were then mixed with varying concentrations of graphene (2.5 and 5.0 mg), and the resulting slurry was ultrasonically treated for 30 minutes. Next, 2-MeIm was gradually added to the prior solution after being dissolved in methanol. The solution that started out dark pink quickly turned dark purple and was stirred for 24 hours at 25 °C. So as to create the finished products, the precipitates were finally recovered by centrifugation. They were rinsed repeatedly with DI–water and then ethanol, following which they were further dried at 80 °C for 24 h. The different samples were labeled as Co-M, Co-M/G2.5 and Co-M/G5. The schematic production procedure of the nanocomposites is shown in Fig. 10(a). The nanocomposites having different compositions were adopted as the electrode materials, and the Co-M/G5 electrode displayed the maximum capacity, measuring 549.96 F g^−1^, which is attributed to the presence of defects in the structure's architecture allowing electric charge carriers to flow more freely which increases the specific capacity. Inside the voltage operation of 1.7 V, the Co-M/G5 asymmetric cell produced a specific capacity of 50.20 F g^−1^ at 20 mV s^−1^, an energy density value of 8.1 W h kg^−1^, and a power density value of 850 W kg^−1^ spanning 1 A g^−1^. This result indicates that the addition of graphene to CoM enhances CoMG5's surface area, improving the electrolyte ion diffusion and electroactive material contact area, and ultimately raising the specific capacitance. A satisfactory cyclic stability of 78.85% was also demonstrated after 1000 cycles of charging and discharging, proving that the nanocomposite's enhanced supercapacitive performance was due to the synergistic action of graphene and Co-MOF. Electrochemical investigations revealed that the combination of graphene and Co-MOF worked synergistically to enhance the supercapacitive performance of the nanocomposite.

Recently, Safari and Mazloom also observed magnificent energy storage performance in Co + Fe bimetallic MOF filaments decorated over reduced GO nanosheets.^144^ Firstly, they prepared GO using modified Hummers' method and then applied an ultrasonic-assisted solvothermal approach to develop the Co + Fe-MOF electrodes. Then they prepared Co + Fe-MOF/rGO electrodes, using 3 and 5 wt% of rGO, showing spindle-like morphology. The highest specific capacitance of 2069.1 F g^−1^ at a current density spanning 0.5 A g^−1^ was evaluated for 3% Co + Fe-MOF/rGO and after 5000 cycles, it showed excellent stability during cycling with 91.3% capacitance preservation at an elevated current density of 10 A g^−1^. This can be ascribed by the considerable increase in the integrated area of 3% Co + Fe-MOF/rGO as compared to the pristine Co + Fe bimetallic MOF as can be seen from the CV curves, which caused enhancement in the electrochemical properties owing to the coating of rGO over the sample. The asymmetric supercapacitor device exhibited good energy density level of 75.8 W h kg^−1^ at power density level of 700 W kg^−1^ that explains the pseudocapacitive property and is ascribed to the faradaic redox processes on the electrode surface during OH^−^ insertion/extraction. Table 5 displays the results of the calculations for average pore size (*r*_p_), specific surface area (*S*_BET_), and pore volume (*V*_p_). When compared to the pristine MOF, the Co + Fe-MOF/rGO demonstrated increased BET surface area and porosity. The boost in SBET of Co + Fe-MOF/rGO over raw MOF could be attributed to the generation of greater permeability at the point of interaction between rGO and MOF. Because the rGO sheets stack around the permeable spindles of Co + Fe-MOF, *S*_BET_ and *r*_p_ drop as the rGO content increases from 3 to 5%. The results exhibited that the 3% Co + Fe-MOF/rGO might make an intriguing electrode material to feed the energy storage devices.

**Table 5:** Sample texture parameters assessed using BET, and charge transfer resistance (*R*_ct_) and equivalent series resistance (*R*_s_) established using EIS measurements.^144^ Adapted with permission from source: *J. Energy Storage*, 2023, **58**, 106390. Copyright 2023 Elsevier

Active material	*S*_BET_ (m^2^ g^−1^)	*V*_p_ (cm^3^ g^−1^)	*r*_p_ (nm)	*R*_s_ (Ω)	*R*_ct_ (Ω)
Co + Fe-MOF	52.7	0.017	1.32	1.01	23.07
3% Co + Fe-MOF/rGO	149.0	0.054	1.79	0.79	5.03
5% Co + Fe-MOF/rGO	112.0	0.027	1.57	0.93	8.04

**Fig. 11:**
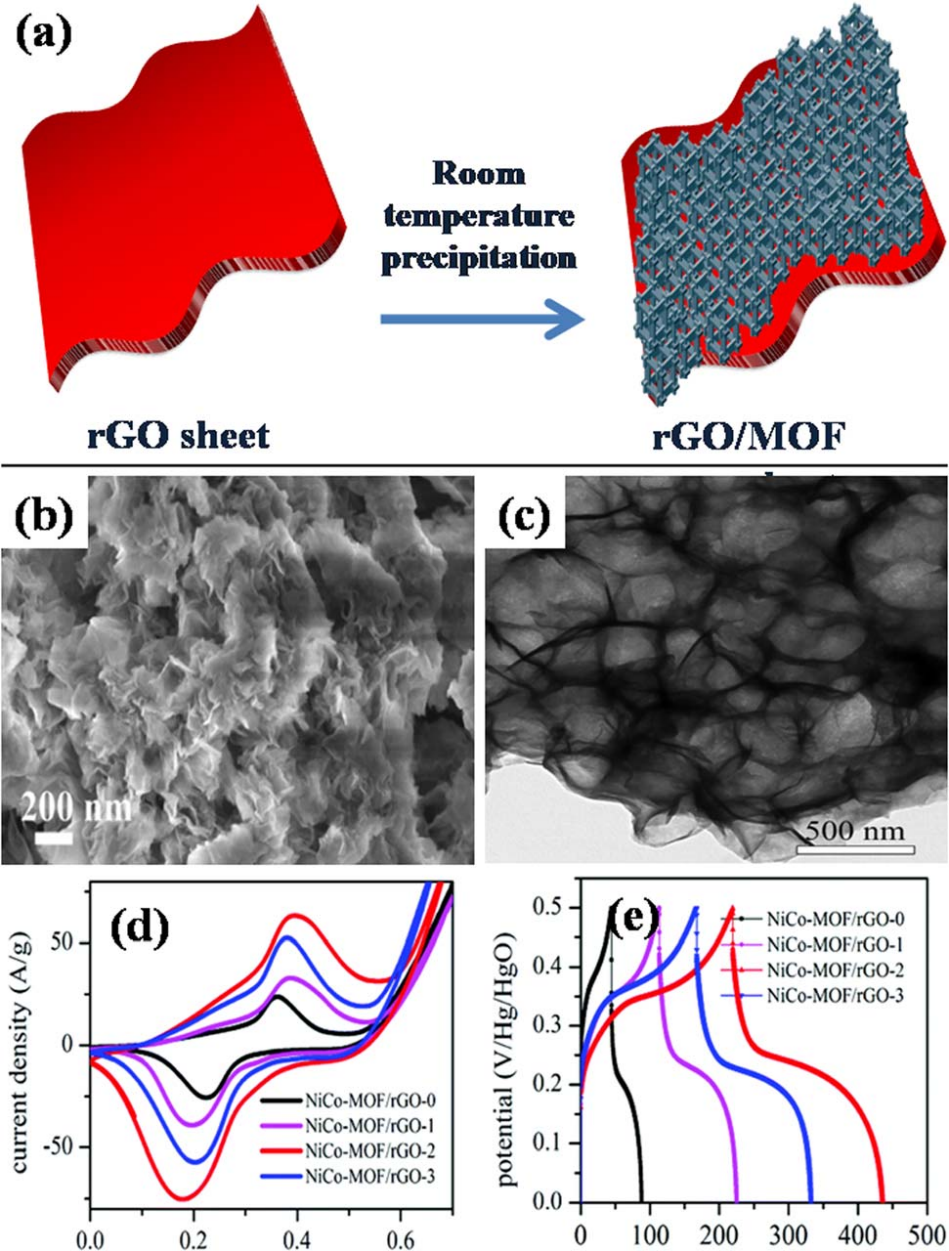
(a) Diagram illustrating the development phase of NCM/rGO through straightforward precipitation at ambient temperature, (b) field effect – scanning electron microscopy image of NCM/rGO2 nano sheets, (c) transmission electron microscope image of NCM/rGO nano sheets, (d) relative cyclic voltammetry plots at scan rate of 1 mV s^−1^ for NCM/rGO2, and (e) relative galvanostatic charge/discharge plots at 4 mA g^−1^ for NCM/rGO2.^145^ (b)–(e) Adapted with permission from source: *RSC Adv.*, 2019, **9**, 36123–36135. Copyright 2019 Royal Society of Chemistry.

A different work reported by Beka's group, in 2019, grew ultrathin NiCo-MOF 2D nanosheets on rGO substrates using conventional room temperature precipitation method.^145^ The growth of nanosheets *via* this route has been illustrated in Fig. 11(a). In specifics, methanol, 2 methylimidazole, cobalt nitrate, nickel nitrate, and the self-made rGO utilizing sulfur as template precursor were combined and blended into a homogeneous solution. The group prepared samples using different concentration of rGO. Thereafter, the solution was allowed to stay at room temperature for a full day. Ultimately, the final product was produced as 2D NiCo-MOF/rGO (NCM/rGO) after centrifugation, washing, and drying. Fig. 11(b) and (c) displays a homogeneous distribution of MOF nanosheets generating a framework resembling a flower, using FE-SEM and TEM analyses, respectively. After adjusting the rGO to MOF ratio, NCM/rGO2 (comprising 10 mg rGO) was used as the best performing hybrid material as compared to other compositions. At a current density spanning 1 A g^−1^, an excellent specific capacitance value of 1553 F g^−1^ was attained for NCM/rGO2. The relative cyclic voltammetry analyses for each of the four samples (NCM/rGO0, NCM/rGO1, NCM/rGO2, and NCM/rGO3) are displayed in Fig. 11(d). The reduction peaks correlate to the reduction of Co^4+^ to Co^3+^ to Co^2+^ and Ni^3+^ to Ni^2+^, whereas the redox peaks were formed during state transition of Co^2+^ to Co^3+^ and Ni^2+^/Ni^3+^. Consequently, the overall oxidation and reduction of peak current densities of both the materials are raised by their various oxidation states. Initially, with the rising quantity of rGO precursor in the sample (NCM/rGO0 < NCM/rGO1 < NCM/rGO2), the CV comparison result revealed that the samples' CV area increased and eventually decreased as the amount of rGO increased (NCM/rGO3 < NCM/rGO2). Relative GCD diagrams of all the samples are illustrated in Fig. 11(e). It was found that all samples' GCD curves show two different phases of discharge: the potential lowers quickly at first, and then it discharges slowly in the later stages. Furthermore, following 5000 cycles of charging and discharging, the sample demonstrated an exceptional cyclic capacity of 83.6%. This may have to do with rGO's benefit as a conducting backbone. Therefore, a spike in the ratio of rGO results in an increase in the hybrid material's conductivity, higher active material consumption, and improved rate capability. Remarkably, the constructed asymmetric gadget exhibited an exceptional energy density level of 44 W h kg^−1^ at a power density level of 3168 W kg^−1^, which is attributed to our porous rGO's exceptional and special qualities, which improve the ionic and electrical conductivity connected to two-dimensional NCS redox nanosheet materials that are active to boost the pseudocapacitive characteristic, and are the primary cause of its exceptional performance. This report's electrochemical performance shows that MOF nanosheet hybridization with carbon materials is a viable strategy for next-gen supercapacitors.

### 5.3 Mixing method

The simple mixing method for synthesizing GO/MOFs involves combining a graphene oxide suspension with MOF crystals through gentle mixing. The process typically includes dispersing GO and MOF in suitable solvents, slowly combining them while stirring to ensure uniform distribution. This preparation technique is able to produce MOF/GO composites in just a few minutes with controlled composition.

**Fig. 12:**
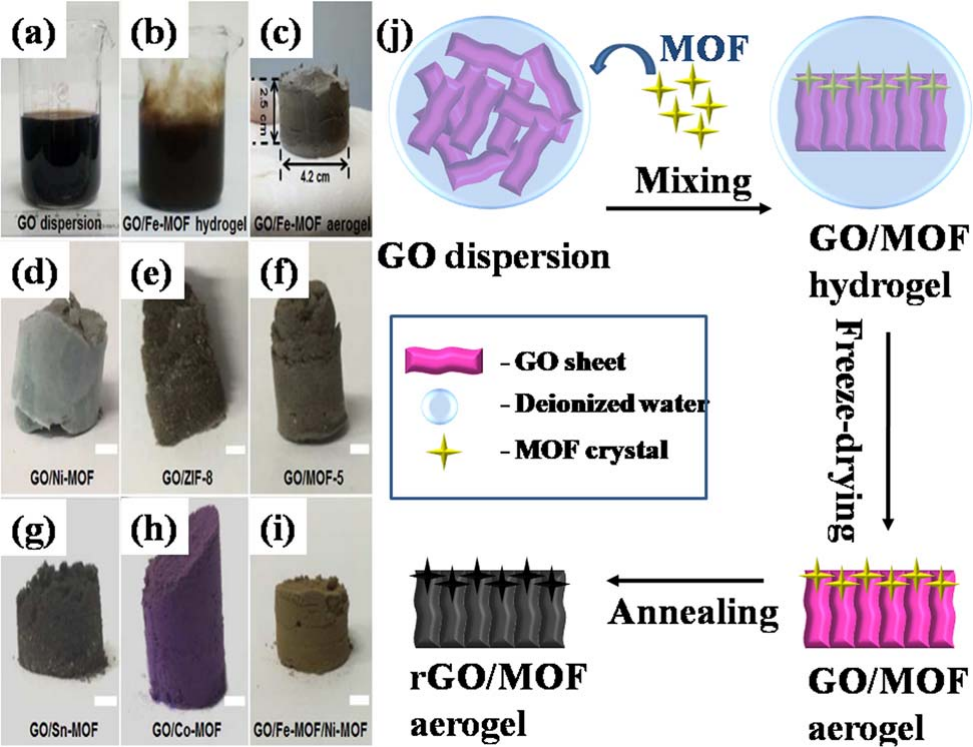
Images showing (a) GO dispersion, (b) iron-MOF/GO hydrogel, (c) iron-MOF/GO aerogel, (d)–(i) additional GO/MOF composite aerogels (d–i, scale bar = 0.35 cm), and (j) a schematic representation of the process of making MOF/GO and MOF/rGO-derived composite aerogels *via* mixing process.^103^ (a)–(i) Adapted with permission from source: *Chem. Mater.*, 2017, **29**, 106390. Copyright 2017 American Chemical Society.

In a paper reported by Xu's group, massive production of several 3D MOF/GO composites was reported utilizing an elementary mixing technique.^103^ The group synthesized the composite in three phases using blended MOF and GO nanosheets. The first stage involved continually stirring MOF and GO to create three-dimensional porous hydrogel nanostructures. The second stage involved freeze-drying the generated hydrogel of the GO/MOF composite in order to immediately transform it into an aerogel-like nanomaterial. Lastly, in step 3, the produced nanomaterials were heated in nitrogen and air atmospheres, respectively, to transform to a pure stage of rGO/MOF. It should be noted that the double-step annealing procedure helps preserve the 3D porous structure and can stop the generated composite aerogels made from rGO and MOF from collapsing. Iron-MOF, nickel-MOF, ZIF8 (Zn cations linked together with 2-methylimidazolate anions), MOF5, tin-MOF, cobalt-MOF, and iron-MOF/nickel-MOF hybrids were among the several composite aerogels created from GO (Fig. 12(a)–(i)) based on this simple and general mixing method illustrated in Fig. 12(j). According to the electrochemical data, at a constant current density spanning 1 A g^−1^, the rGO/Fe_2_O_3_ nanocomposite electrode produced an enormous specific capacitance of levels 869.2 F g^−1^ as compared to rGO (360.8 F g^−1^) and Fe_2_O_3_ (258.5 F g^−1^), respectively. Primarily, the 3D networks comprising interconnected reduced rGO sheets contribute significantly to the substantial electric double-layer capacitance and exceptional electrical conductivity. Secondly, the structures composed of Fe_2_O_3_ derived from MOFs are comprised of numerous Fe_2_O_3_ nanoparticles, each measuring less than 10 nm. These nanoparticles exhibit a heightened concentration of active surface area, thereby imparting faradaic capacitance to the composite material.

**Fig. 13:**
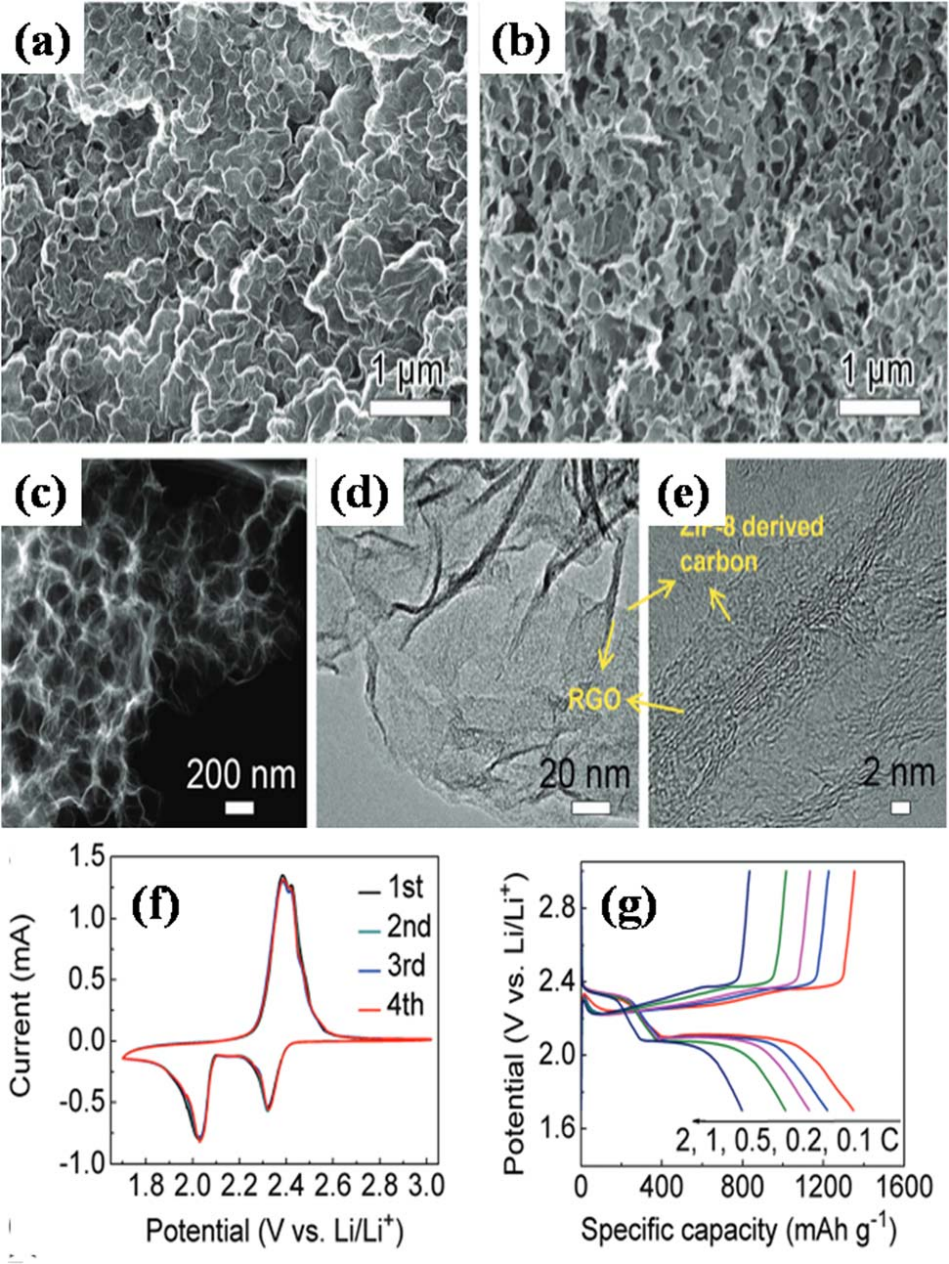
Morphologies of the PCF: (a) and (b) scanning electron microscope images, (c) scanning transmission electron microscope image, (d) transmission electron microscope image, (e) high resolution – transmission electron microscope image, (f) cyclic voltammetry curves spanning a scan rate of 0.1 mV s^−1^, and (g) galvanostatic charge/discharge profiles.^146^ (a)–(g) Adapted with permission from source: *Small Methods*, 2019, **3**, 106390. Copyright 2019 Wiley Online Library.

In a different report, Ding *et al.* used MOF particles of zeolitic imidazolate frameworks (ZIF-8) with GO nanosheets to fabricate the composite structure *via* mixing.^146^ The positively charged ZIF polyhedra (zeta potential, *V*_*z*_ = +3.1 mV) were dynamically enveloped by the negatively charged nanosheets of GO (*V*_*z*_ = −45.8 mV). The solution's *V*_*z*_ dropped to −7.3 mV when the two were incorporated together, suggesting that a portion of the charge is neutralized by the interaction involving ZIF and GO. The hybrid framework of ZIF/GO composite was created following vacuum filtering. Pyrolyzing the structure in question generated a three-dimensional carbon porous framework (PCF) consisting of polyhedral vacuous carbon plated with reduced GO, as can be seen in Fig. 13(a)–(e). The hybrid electrode exhibited a substantial discharge ability of 1151 mA h g^−1^, and after 650 cycles, it demonstrated a modest capacity collapse of 0.035% per cycle. Several potentially synergistic features in the design of the PCF/S electrode contribute to its remarkable cycling efficiency. Firstly, the utilization of highly conductive and mechanically robust graphene facilitates the deposition of sulfur and enables the efficient transfer of electrons between layers within the active material. Secondly, the volume expansion of the sulfur composite during charge and discharge phases is accommodated, and the accessibility of the electrolyte to sulfur is enhanced through the geometrically structured channels inherent in the three-dimensional carbon scaffolding. Fig. 13(d)–(f) shows the cyclic voltammetry and galvanostatic charge/discharge profiles for the synthesized composites. Therefore, by offering straightforward, scalable methods to create 3D porous designs with heterostructured interfaces, these materials can be a promise in the field of energy storage avenues.

### 5.4 Mechanical shear blending with scribing

**Fig. 14:**
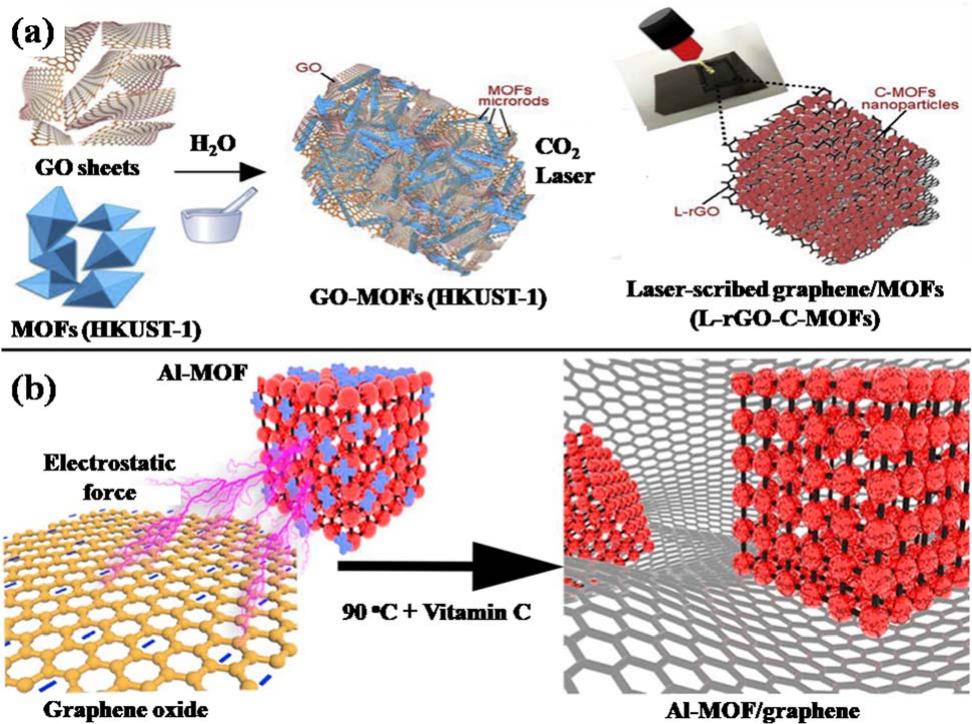
(a) The schematic diagram illustrates the preparation process of the GO-MOFs (HKUST-1) composite. It entails mechanically blending MOFs and GO in an aqueous solution, followed by laser treatment (scribing), resulting in the formation of a highly porous 3D nanostructured composite utilized as an electrode in supercapacitors and (b) a schematic illustrating the preparation process of the Al-MOF/graphene composite is depicted, where positive and negative charges are denoted by the symbols “+” and “−“, respectively, on the surfaces of Al-MOF and graphene oxide. (a) Adapted with permission from source: *Electrochim. Acta*, 2020, **329**, 135104. Copyright 2020 Elsevier. (b) Adapted with permission from source: *Nano Energy*, 2019, **65**, 104032. Copyright 2019 Elsevier.

Mechanical shear blending followed by scribing is a method used to produce nanocomposite materials with controlled dispersion and alignment of nanofillers (GO) within the MOF matrix. A novel porous 3D-structured carbon composite material, incorporating carbonized metal–organic framework (C-MOF) microrods derived from HKUST-1, and graphene, was developed by Van *et al.* for high-performing supercapacitors.^147^ The combination of HKUST-1 microrods' 1D structure and graphene's 2D structure yields a composite material with enhanced electron and charge transportation capabilities. The proposed concept depicted in Fig. 14(a) is based on two innovative strategies. Firstly, to achieve a unique three-dimensional composite structure, HKUST-1 microrods and GO were combined using a straightforward synthetic technique known as mechanical grinding. Upon combining these two components in an aqueous shear mixing environment, the HKUST-1 fragments or seeds rapidly grew into rods with diameters ranging from nano-to-microns. The uniformly dispersed HKUST-1 microrods act as spacers within the GO matrix, preventing agglomeration and restacking of GO sheets, which is crucial for the formation of robust three-dimensional structures. Additional processing of the composite film was carried out to enhance its conductive properties, enabling it to function effectively as an electrode for supercapacitors. In this subsequent stage, GO was transformed into the conductive and porous material known as laser-induced reduced graphene oxide (L-rGO) using CO_2_ laser scribing to produce a film. After 5000 cycles, the produced film exhibited a cyclic stability of 97.8% at 10 A g^−1^ and a high capacitance of 390 F g^−1^ at 5 mV s^−1^. Furthermore, with an exceptional energy density of 22.3 W h kg^−1^ and an outstanding power density of 8037.5 W kg^−1^, the symmetrical supercapacitor validated a new pathway for fabricating 3D porous graphene-MOF composites for high-performance energy storage devices. This approach differs from traditional methods of converting GO to rGO, which typically involve heat or chemical reduction, processes that may damage or degrade the delicate HKUST-1 microrods.

### 5.5 *Ex situ* synthesis

*Ex situ* approach enables the synthesis of MOF and graphene composites by incorporating specific chemicals. The synthesis process may be time-consuming, and the *ex situ* approach could potentially alter the characteristics of the composite material. While the qualities of the two substances being combined and their interaction must meet certain conditions, the direct mixing approach is easier to manipulate. Following this approach, Gao *et al.* synthesized Al-MOF particles and thereafter graphene was uniformly coated onto them to form the Al-MOF/graphene composite.^148^ Subsequently, the composite underwent lithiation and delithiation processes, leading to a significant structural alteration of the Al-MOF particles characterized by an order–disorder transition (Fig. 14(b)). This transition facilitated the diffusion and storage of Li^+^ ions by creating more open channels. At a current density of 100 mA g^−1^, the composite exhibited a continuous increase in capacity from 60 to 400 mA h g^−1^. The remarkable enhancement observed in Li^+^ ion migration and storage can be attributed to the lithiation/delithiation-induced structural disordering in MOF crystals, which creates additional channels for ion transport. The presence of graphene sheets enables the MOF particles to maintain their electrical conductivity with the current collector. Moreover, these sheets facilitate charge transfer processes and Li^+^ ion migration kinetics across the Solid Electrolyte Interphase (SEI) layer, thereby enhancing electronic conductivity. The study suggests that enhancing the capacity and cycling stability of anode materials requires both the lithiation/delithiation-induced order–disorder transition in MOFs and the optimal encapsulation of MOF by graphene.

## 6 Characterization techniques

### 6.1 Structural and morphological characterizations

The structural and morphological characterization of MOF/GO composites involves various analytical techniques to understand their composition, crystal structure, morphology, and surface features.

**Fig. 15:**
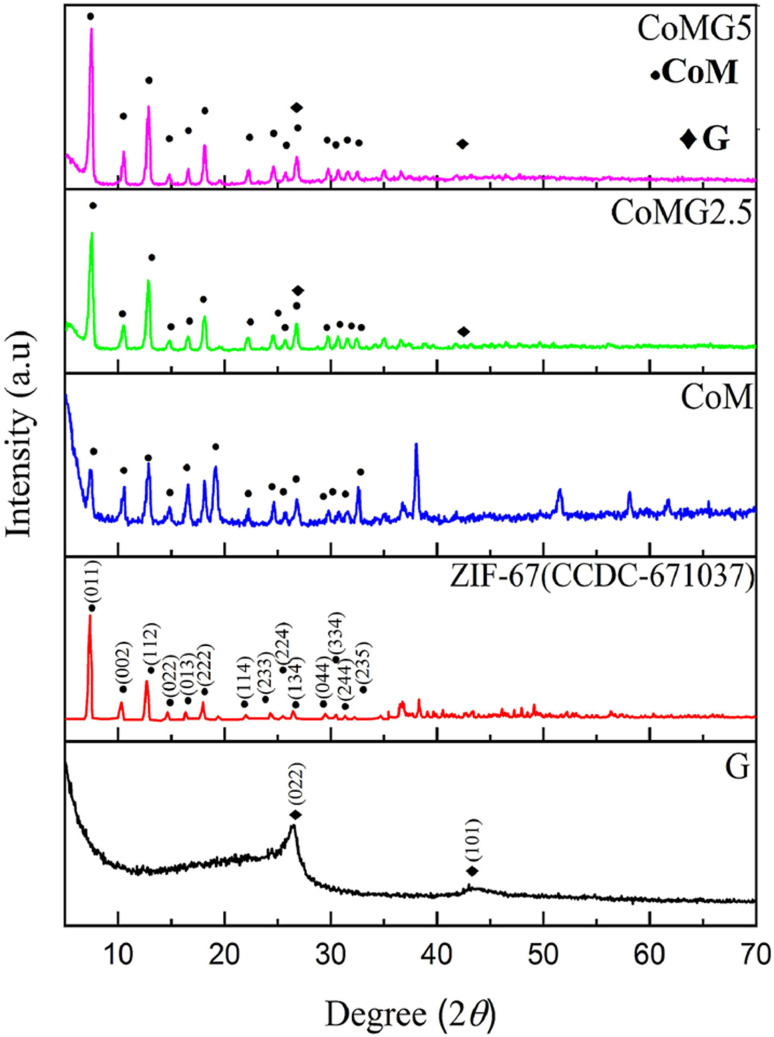
Powder X-ray diffraction patterns of G, Co-M, Co-M/G2.5 and Co-M/G5 samples.^110^ Adapted with permission from source: *J. Energy Storage*, 2021, **33**, 101925. Copyright 2021 Elsevier.

One of the common techniques employed for this purpose is X-ray diffraction. XRD is an effective tool for establishing crystal structure of materials. By subjecting the MOF/GO composite to X-rays, the arising diffraction pattern can be analyzed to scrutinize the crystalline phases that are existing and obtain information about the unit cell parameters, crystallite size, and preferred orientation. Azadfalah's group used a facile one-step precipitation method to create Co-MOF/graphene nanocomposites.^110^ They employed XRD for the analysis of graphene (G) with different compositions of ZIF-67, namely, Co-M, Co-M/G2.5, and Co-M/G5. As we can see in Fig. 15, two diffraction peaks (2 *θ* = 26.5° and 2 *θ* = 43°) were visible in the XRD pattern of G. These peaks matched JCPDS no. 0284-25 and corresponded to reflections originating from the graphitic structure in accordance with (002) and (101) planes, respectively. The peaks in the Co-M XRD pattern at 2 *θ* = 7.44°, 10.56°, 12.92°, 14.89°, 16.55°, 18.13°, 22.18°, 24.66°, 25.22°, 26.83°, 82.29°, 30.70° and 32.63° corresponded, respectively, to the reflections arising from (011), (002), (112), (022), (013), (222), (114), (233), (224), (134), (044), (334), (244), and (345) planes, showing that there is good agreement between the XRD pattern of the ZIF-67 single crystal and the diffraction pattern of the synthesized Co-M. The Debye-Scherrer equation yielded crystallite sizes of 25, 18, and 22 nm for the Co-M, Co-M/G2.5, and Co-M/G5 samples, respectively. Evidently, the inclusion of graphene is the cause of this size reduction.

To further characterize the morphology of the synthesized composites visually, Scanning Electron Microscopy (SEM) and Transmission Electron Microscopy (TEM) were employed. SEM and TEM provide high-resolution images of the surface and internal structure of materials. These techniques can reveal information about the morphology, particle dimensions, and spatial arrangement of MOF nanoparticles on the graphene oxide sheets.

**Fig. 16:**
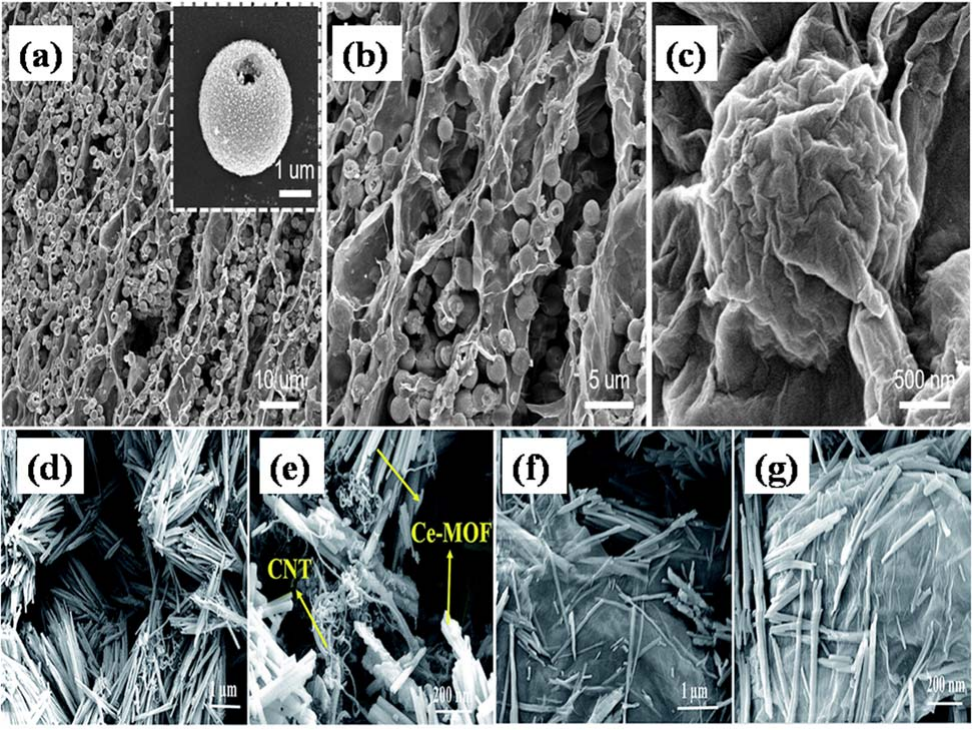
Scanning electron microscope images of (a)–(c) the graphene nanosheets completely encapsulated and adhered to the Ni-MOFs under diverse magnifications, which achieves a 3D porous architecture, (d) and (e) uniform sized nanorod structure of CeMOF@CNT composites, (f) and (g) uniform sized nanorod structure of CeMOF@GO composites.^113^ (a)–(g) Adapted with permission from source: *RSC Adv.*, 2018, **8**, 3462–3469. Copyright 2018 Royal Society of Chemistry.

SEM allows researchers to observe the surface morphology and topography of MOF/GO composites at high resolution. It provides insights into the particle size, distribution, and the overall structure of the composite. Zong's group, recently in 2023, developed 3-D porous aerogels that had superior capacitive characteristics for adaptable energy storage by embedding open-hollow Ni-MOF microspheres onto rGO nanosheets.^149^ SEM analysis revealed that the prepared composites had a rough surface, an average diameter of around 2 μm, and an open-hollow structure with a homogeneous spherical shape. Moreover, the graphene nanosheets were completely wrapped and attached to the composite surface, as seen in Fig. 16(a)–(c), resulting in a 3D porous architecture. The pace of electron/ion transport can be greatly accelerated by both graphene nanosheets and composites, and the resulting three-dimensional porous architecture can greatly reduce the length of ion dissemination pathways, increase the amount of specific surface area, and provide an abundance of exposed sites for activity. In a different report by Ramachandran *et al.*, wet chemical synthesis of CeMOF@GO and CeMOF@CNT composites was carried out at room temperature.^113^Fig. 16(d)–(g) shows their SEM images. The resulting CeMOFs in GO and CNT are evenly scaled nanorod structures with a length of few micrometers and a diameter of about 150 nm, as demonstrated by the SEM pictures. In addition to serving as spacers, the deposited nanorods can also prevent GO sheets from stacking again. Conversely, the GO sheets may aid in stopping the Ce-MOF nanorods from clumping together.

**Fig. 17:**
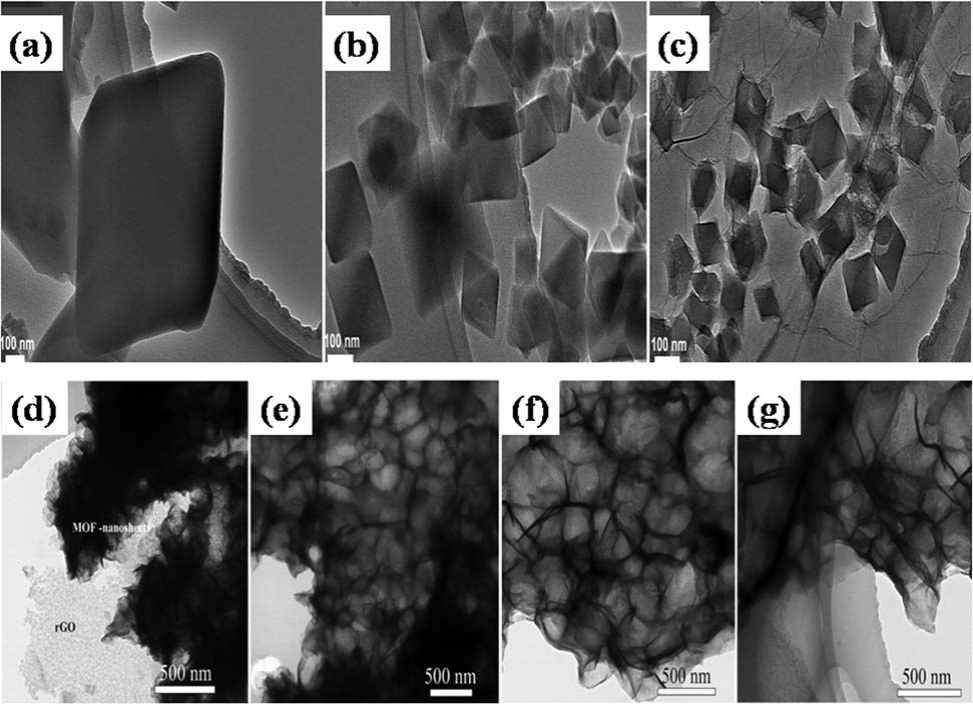
Transmission electron microscope micrographs of (a) CrMOF, (b) CrMOF@PAMAM, (c) CrMOF/GO@PAMAM showing regular octahedrons with excellent crystallinity,^140^ and (d) to (g) transmission electron microscope images of Ni + Co/MOF@rGO nanosheets at different magnification exhibiting the translucent GO blending with ZIF particles where its excellent flexibility is made apparent by the presence of creases and folds.^145^ (a)–(c) Adapted with permission from source: *ACS Omega*, 2021, **6**, 31184–31195. Copyright 2021 American Chemical Society, and (d)–(g) Adapted with permission from source: *RSC Adv.*, 2019, **9**, 36123–36135. Copyright 2019 Royal Society of Chemistry.

TEM provides even higher resolution images than SEM, enabling researchers to visualize the internal structure of MOF/GO composites, including the arrangement of nanoparticles, layers, and any defects present. A nanocomposite was formed by combining chromium-centered MOF nanoparticles and GO using a simple ultrasonication method by Cui *et al.*, which was governed by the dendrimerpolyamidoamine (PAMAM).^140^ TEM pictures of CrMOF, CrMOF@PAMAM, and CrMOF/GO@PAMAM nanocomposites show their textures in Fig. 17(a)–(c). The micrographs show a substantial quantity of well-crystallized regular octahedrons. The CrMOF@PAMAM images demonstrated some pronounced wrinkles consistent with GO's properties, evenly coated across the surface of CrMOF. This showed that the morphology had been well-maintained, creating certain three-dimensional hole formations. In other report, Beka's group prepared a Ni + Co/MOF@rGO hybrid material using a level precipitation procedure at ambient temperature.^145^Fig. 17(d)–(g) the TEM pictures of Ni + Co/MOF@rGO hybrid material which make it evident how rGO sheets are formed onto ultrathin MOF nanosheets. It is amazing how its excellent elasticity is revealed by the creases and folds.

Besides these techniques, thermogravimetric analysis (TGA) is also employed by researchers, which measures the weight changes of a material as a function of temperature. It can be used to scrutinize the thermal stability of MOF/GO composites and ascertain the content of different components based on their decomposition temperatures.

### 6.2 Electrochemical characterizations

**Fig. 18:**
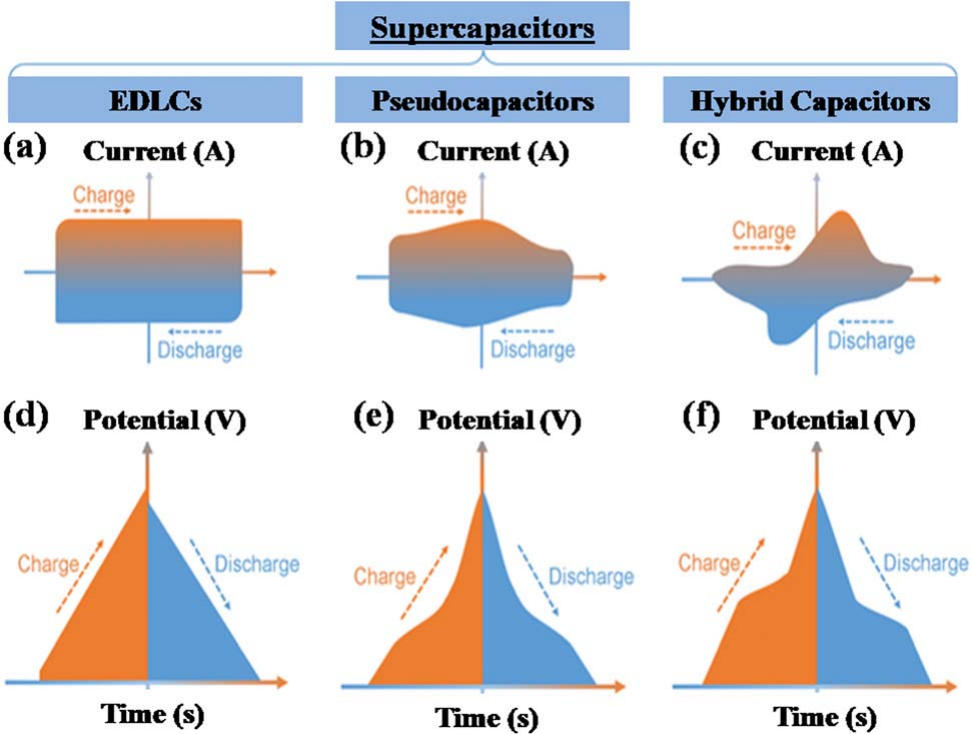
A comparison of the electrochemical behavior of conventional supercapacitors can be illustrated through cyclic voltammetry (CV) and galvanostatic charge–discharge (GCD) curves, as shown in panels (a) and (d) for electric double-layer capacitors (EDLCs), panels (b) and (e) for Pseudocapacitors, and panels (c) and (f) for hybrid capacitors, respectively. (a)–(f) Adapted with permission from source: *Adv. Funct. Mater.*, 2022, **32**, 2108107. Copyright 2022 Wiley Online Library.

Electrochemical characterizations play an essential role in assessing the effectiveness of MOF/GO composites for various applications. The results obtained from these techniques contribute to optimizing the design and performance of MOF/GO composites for diverse electrochemical applications. Fig. 18 shows the comparison between CV and GCD curves of different types of supercapacitors.^150^ Here's how each technique is applied to different types of supercapacitors.

#### 6.2.1 Cyclic voltametry (CV)

CV is a widely used electrochemical technique that provides information about the redox behavior and electrochemical stability of MOF/GO composites. By applying a potential sweep, CV generates current–voltage curves that can reveal details about the electrochemical processes, capacitance, and the presence of specific redox couples. The following formula could be used to get an electrode's specific capacitance (*C*) from cyclic voltammetry:10
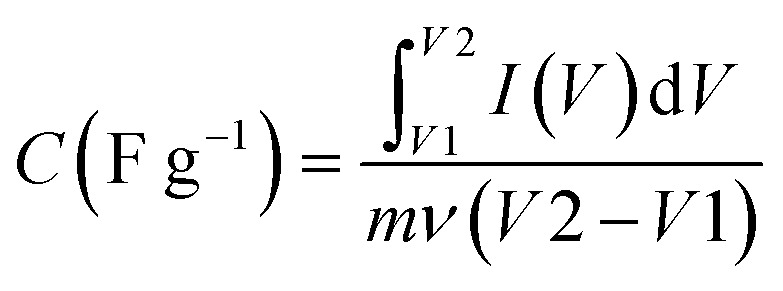


In the provided context, where *C* represents the specific capacitance (in F g^−1^), *m* is the mass of the active electrode (in g), *ν* is the scan rate (in V s^−1^), and *V*1 and *V*2 are the potential limits (in *V*), the expression 
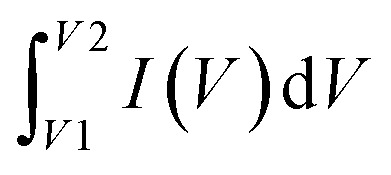
 signifies the area under the cyclic voltammetry (CV) curve within the specified voltage range. Here's an outline discussing the cyclic voltammetry characterization for different types of supercapacitors.

(a) Electrochemical Double-Layer Capacitors (EDLCs): EDLCs typically exhibit rectangular-shaped CV curves, indicating ideal capacitive behavior. The area under the CV curve represents the charge storage capacity of the supercapacitor.

(b) Pseudocapacitors: pseudocapacitors exhibit non-linear CV curves due to faradaic redox reactions occurring at the electrode surface. The curves may show peaks or humps corresponding to redox processes.

(c) Hybrid capacitors: hybrid capacitors may exhibit a combination of capacitive and faradaic behavior in the CV curves, reflecting the contribution from both EDLC and pseudocapacitive components.

#### 6.2.2 Galvanostatic charge/discharge (GCD)

GCD is commonly employed to investigate the energy storage performance of MOF/GO composites, particularly in supercapacitors and batteries. By applying a constant current, GCD measures the charging and discharging characteristics, providing information on capacitance, charge storage, and overall electrochemical performance. The following formula can be utilized to determine the specific capacitance from the galvanostatic charge–discharge (GCD) investigation:11
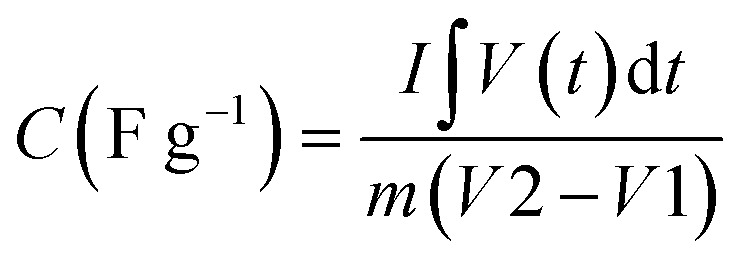


In the provided equation, where *I* represents the steady discharge current during GCD measurement, *m* is the mass of the active electrode (in g), 
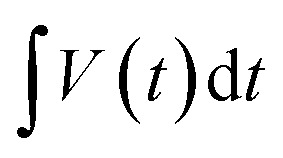
 denotes the area under the discharge curve, and (*V*2 − *V*1) represents the working potential window (in *V*).

One can compute a supercapacitor's energy density (*E*) and power density (*P*) using the following formula:12
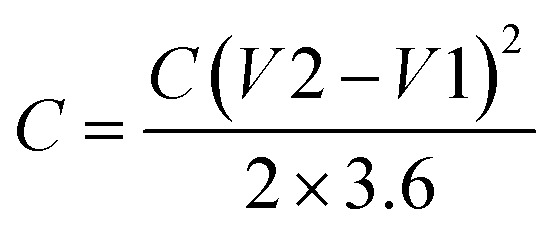
and13
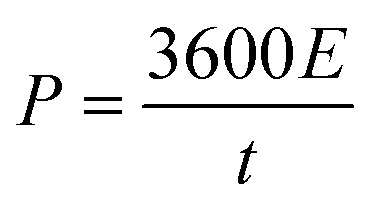


Here's an outline discussing the galvanostatic charge/discharge characterization for different types of supercapacitors.

(a) Electrochemical Double-Layer Capacitors (EDLCs): GCD curves for EDLCs are linear, reflecting the capacitive behavior with a constant slope. The slope of the discharge curve provides information about the equivalent series resistance (ESR) of the supercapacitor.

(b) Pseudocapacitors: GCD curves for pseudocapacitors may exhibit non-linear behavior, with voltage plateaus or slopes corresponding to the redox reactions in addition to the capacitive behavior.

(c) Hybrid capacitors: GCD curves for hybrid capacitors may show a combination of linear behavior and non-linear features corresponding to both capacitive and faradaic processes.

#### 6.2.3 Electrochemical impedance spectroscopy (EIS)

EIS is a technique used to study the electrical properties and impedance of MOF/GO composites. By applying a small alternating current at different frequencies, EIS provides information on the resistive and capacitive components of the composite, helping to understand the charge transfer processes, interfacial resistance, and overall electrochemical behavior. Here's an outline discussing the electrochemical impedance spectroscopy characterization for different types of supercapacitors.

(a) Electrochemical Double-Layer Capacitors (EDLCs): EIS of EDLCs typically shows a semicircle in the high-frequency region, representing the charge transfer resistance at the electrode–electrolyte interface. At low frequencies, a linear portion corresponds to diffusion-controlled processes in the electrolyte.

(b) Pseudocapacitors: EIS of pseudocapacitors may show additional features such as Warburg impedance, indicating the diffusion of ions within the electrode material. The presence of faradaic processes introduces additional elements in the impedance spectrum.

(c) Hybrid capacitors: EIS of hybrid capacitors may reveal a combination of semicircles, linear portions, and additional features corresponding to both charge transfer resistance and ion diffusion processes associated with EDLC and pseudocapacitive components.

**Fig. 19:**
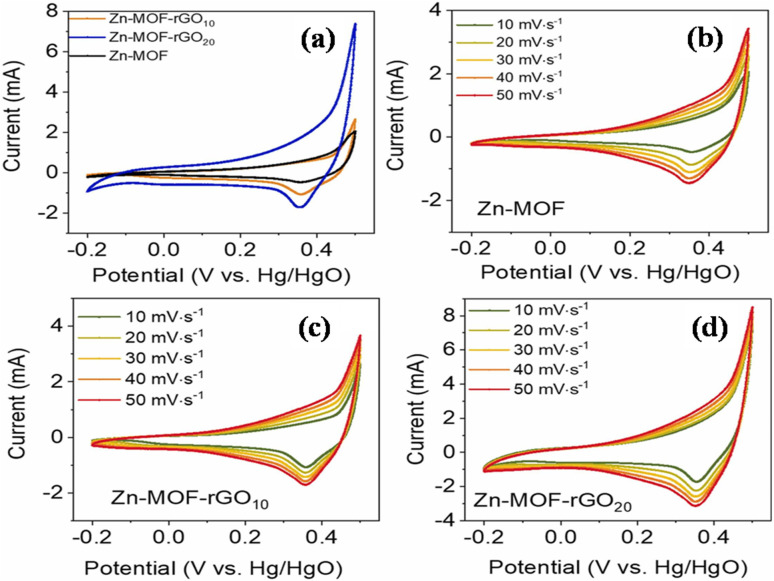
(a) ZnMOF, ZM@rGO10, and ZM@rGO20 cyclic voltammetry profiles at a 10 mV s^−1^ scan rate; patterns of cyclic voltammetry monitoring at different scan rates of (b) ZnMOF, (c) ZM@rGO10, and (d) ZM@rGO20.^111^ (a)–(d) Adapted with permission from source: *Synth. Met.*, 2022, **290**, 117155. Copyright 2022 Elsevier.

Thi *et al.*, in 2022, synthesized a low-cost nanocomposite of Zn-MOF-rGO (ZM@rGO) by hydrothermal technique which depicted a porous wrinkled nanosheet-like network.^111^ They prepared two types of composites on account of the rGO's concentration: ZM@rGO10 and ZM@rGO20. The electrochemical properties of Zn-MOF, ZM@rGO10, and ZM@rGO20 composite electrodes were ascertained by employing a three-electrode setup in a 3 M aqueous KOH electrolyte for CV, GCD, and EIS investigations. With a 10 mV s^−1^ scan rate, the Zn-MOF, ZM@rGO10, and ZM@rGO20 nanocomposites' CV plots are displayed in Fig. 19(a) inside the potential zone of −0.2 V and +0.5 V. The redox peaks of all three electrodes are clearly apparent on the CV curves, suggesting that the faradaic reaction is occurring as the procedure is being carried out. It's important to note that, out of the three CV curves, the ZM@rGO20 curve had the largest area, indicating that it performs better electrochemically than Zn-MOF and ZM@rGO10. Additionally, the CV curves demonstrated a wide range of interaction between extremely reactive material sites and electrolytic ions, suggesting that the surface faradaic redox reaction may be primarily responsible for the materials' capacitance properties. The results are consistent with a recurring observation regarding MOF-based supercapacitors' hybrid battery-like and pseudocapacitive-like functionality.^151–153^ Cyclic voltammetry (CV) profiles for ZnMOF, ZM@rGO10, and ZM@rGO20 electrodes in Fig. 19(b)–(d) demonstrate increased integrated CV curve areas at higher scan rates (10–50 mV s^−1^), indicating improved electrochemical performance for all three electrodes by the addition of GO to ZnMOF in certain amount.

**Fig. 20:**
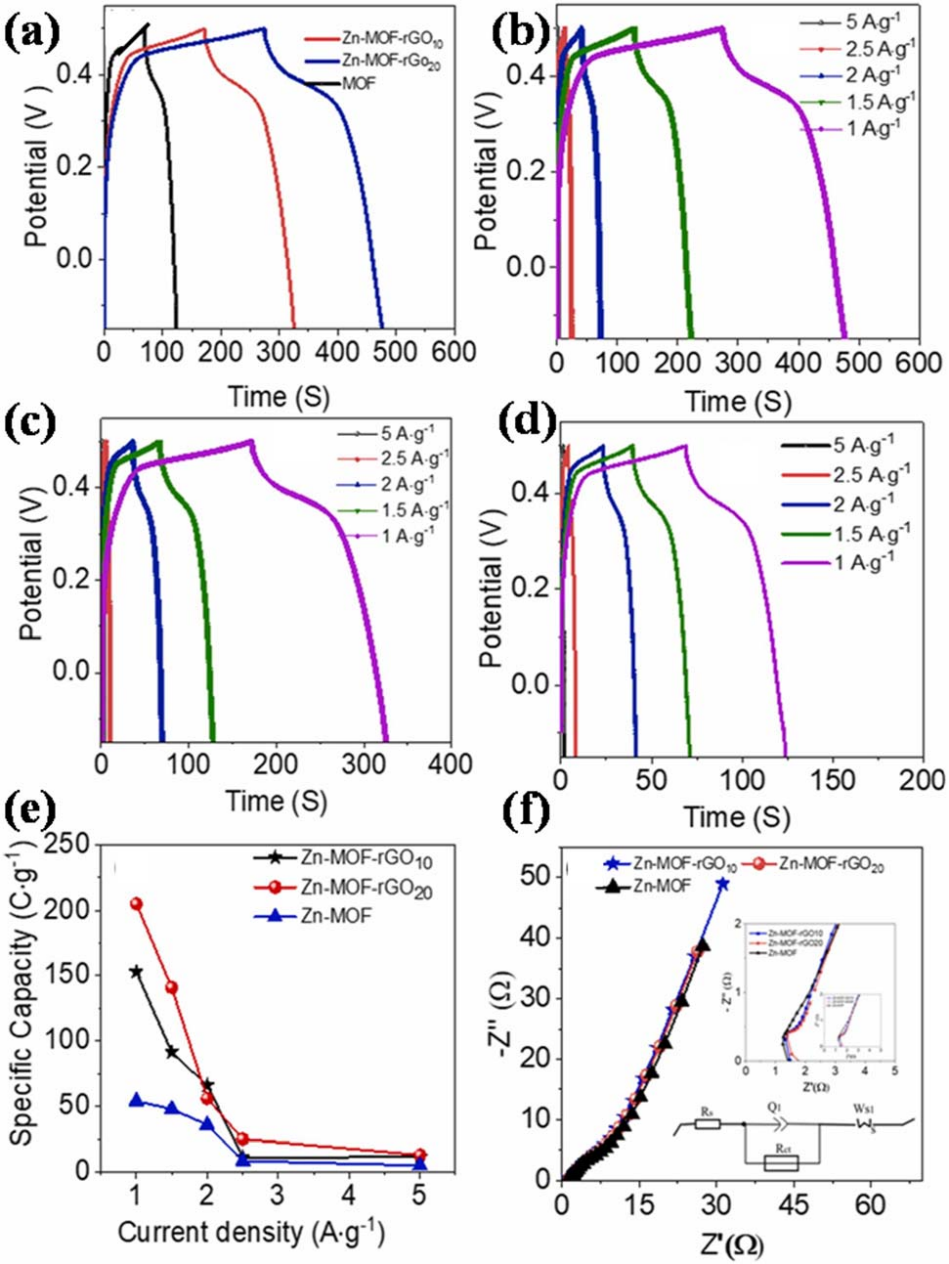
(a) Galvanostatic charge/discharge curves for ZnMOF, ZM@rGO10, ZM@rGO20 at a current density of 1 A g^−1^, (b) galvanostatic charge/discharge plots for ZM@rGO20, (c) ZM@rGO10, (d) ZnMOF at various current densities, (e) graph of specific capacity *versus* current density, and (f) electrochemical impedance spectra (EIS) (enlarged view image of Nyquist plot and connected circuit of EIS shown in the inset).^111^ (a)–(f) Adapted with permission from source: *Synth. Met.*, 2022, **290**, 117155. Copyright 2022 Elsevier.

The galvanostatic charge–discharge (GCD) curves for ZnMOF, ZM@rGO10, and ZM@rGO20 at a current density of 1 A g^−1^ are illustrated in Fig. 20(a) to assess the electrochemical behavior within the potential operation of −0.15 to 0.5 V. In comparison to ZM@rGO10 and pristine ZnMOF, it is evident that the ZM@rGO20 electrode has achieved a longer discharge time, which suggests that the functionality of the supercapacitor is considerably affected by the appropriate amount of rGO in pure ZnMOF. Fig. 20(b)–(d) shows graphical representations depicting the GCD contours for all the electrodes estimated for the current density levels that fall between 1–5 A g^−1^ region, wherein each of the three electrodes' voltage lines shows a unique plateau area beneath the current density, and their very symmetrical shape indicates significant reversibility. Additionally, ZnMOF and the composites ZM@rGO10 and ZM@rGO20 were subjected to electrochemical impedance spectroscopy (EIS) (Fig. 20(e)) and the Nyquist plot, along with the area inset figure that corresponds to a connected circuit, is displayed in Fig. 20(f). ZM@rGO20 exhibits a lower charge transfer resistance (*R*_ct_) of 1.23 Ω cm^−2^, which can be attributed to the rapid rate of reactions amongst the active electrode and electrolyte than ZM@rGO10 (1.7 Ω cm^−2^) and ZnMOF (1.85 Ω cm^−2^). Notably, the decreased resistance value also shows that KOH electrolytes and active electrodes are well suited to one another.

**Fig. 21:**
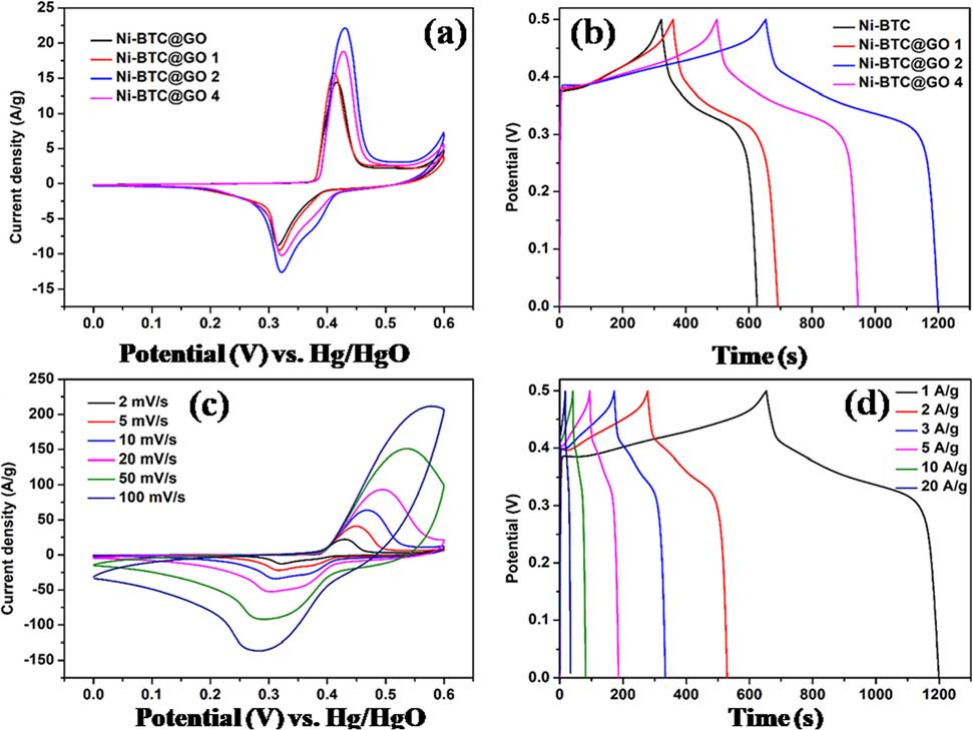
(a) Cyclic voltammetry curves of NiBTC and NiBTC@GO composites spanning 2 mV s^−1^, (b) galvanostatic charge/discharge curves of NiBTC and NiBTC@GO composites spanning 1 A g^−1^, (c) galvanostatic charge/discharge curves of NiBTC@GO2 spanning 2–100 mV s^−1^, and (d) galvanostatic charge/discharge curves of NiBTC@GO2 spanning 1–20 A g^−1^.^154^ (a)–(d) Adapted with permission from source: *ACS Omega*, 2023, **8**, 10888–10898. Copyright 2023 American Chemical Society.

Recently, in 2023, Chen's group presented a newly developed *in situ* synthesis technique for GO/MOF composites (Ni-BTC/GO) for high-performance supercapacitors.^154^ Ni-BTC/GO 0, 1, 2, and 4 are the series hybrid materials that were developed by altering the GO dosage (0, 0.01, 0.02, and 0.04 g of GO). Their group also conducted CV, GCD and EIS analyses using a system of three electrodes in a 3 molar KOH solution to assess the electrochemical capacitance outcomes. According to the tests, GO had a specific capacitance value of 180 F g^−1^, which can be ascribed to the characteristic pseudocapacitive behavior, which stems from the reversible Faraday reaction caused by Ni^2+^ in alkaline solution, showing distinct redox peaks (Fig. 21(a)) instead of rectangular forms. When NiBTC@GO2 is contrasted with alternative electrodes, a larger peak current is produced, indicating a considerable increase in electrochemical activity. Fig. 21(a) depicts the integrated area under the CV curves for all electrodes in order: NiBTC < NiBTC@GO1 < NiBTC@GO4 < NiBTC@GO2. This order also corresponds to the specific capacitance levels of the electrodes, which is further supported by the GCD curves spanning 1 A g^−1^ in Fig. 21(b), which demonstrate that NiBTC@GO2 offers a prolonged charging and discharging duration. Furthermore, each and every GCD curve clearly shows charging and discharging plateaus, illustrating the characteristic pseudocapacitive behavior. The NiBTC@GO2 CV curves are displayed in Fig. 21(b) wherein it is observed that more prominent redox peaks are seen in NiBTC@GO2, indicating a significant increase in capacitance owing to the synergistic effects between Ni-BTC and GO. For current density ranging between 1–20 A g^−1^, the GCD values of NiBTC@GO2 were measured (Fig. 21(d)). The perceived potential region spanning 0 and 0.5 V in the GCD slopes of NiBTC@GO2 illustrates typical pseudocapacitive behavior. Good coulombic efficiency is demonstrated by NiBTC@GO2, as seen by the symmetrical form of the arcs throughout the quick charge–discharge operation. NiBTC, NiBTC@GO1, NiBTC@GO2, and NiBTC@GO4 have specific capacitances of 625.6, 691.6, 1199, and 945.4 F g^−1^, respectively. By shortening the ion transport channel and raising the specific capacitance, layers of GO on the exterior can greatly increase the conductive properties of Ni-BTC. The charging and discharging times increase first, then decrease as the amount of GO increases. This indicates that the GO level in NiBTC@GO1 is unsaturated, which is why it has a lower specific capacitance and conductivity than NiBTC@GO2. However, an overabundance of GO in NiBTC@GO4 would result in worsening GO agglomeration and reduced conductivity performance. NiBTC@GO2 shows up more prominent redox peak levels, indicating a significant increase in capacitance. The specific capacitance levels recorded for NiBTC@GO2 are 1199, 1058.6, 1000.8, 927, 815, and 676 F g^−1^ at 1, 2, 3, 5, 10, and 20 A g^−1^, respectively.

**Fig. 22:**
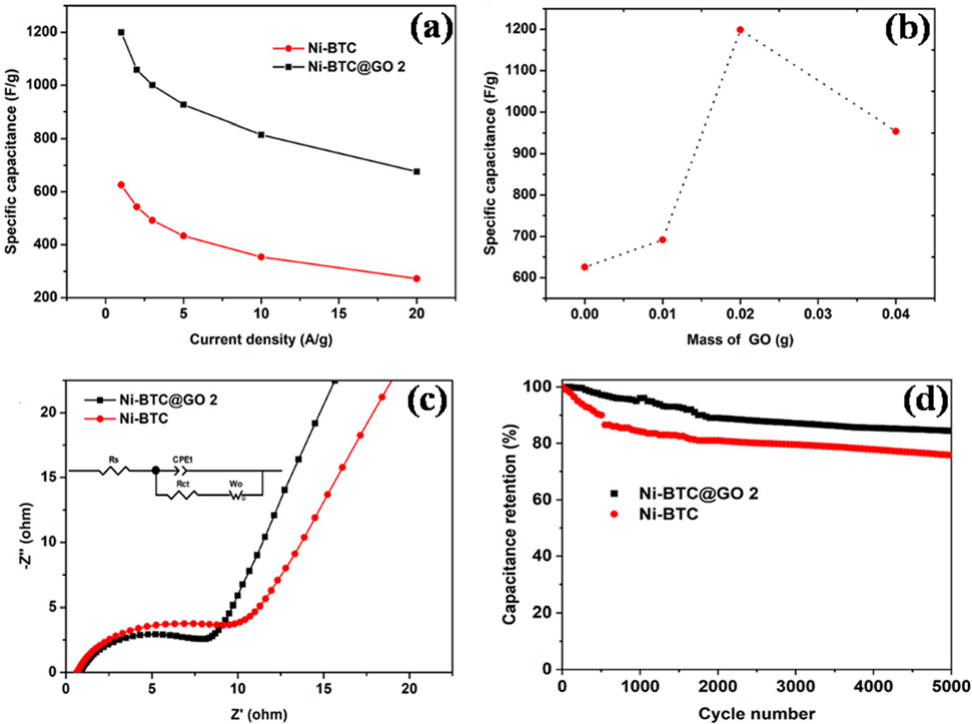
(a) Specific capacitance of NiBTC and NiBTC@GO2 spanning various levels of current densities, (b) specific capacitance as a function of GO mass at a current density of 1 A g^−1^, (c) NiBTC and NiBTC@GO2 electrochemical impedance spectroscopy (EIS) plots in the frequency range of 0.01 Hz to 100 kHz, and (d) performance of NiBTC and NiBTC@GO2 cycling in 3 M KOH electrolyte spanning 1 A g^−1^ of current density.^154^ (a)–(d) Adapted with permission from source: *ACS Omega*, 2023, **8**, 10888–10898. Copyright 2023 American Chemical Society.

Nyquist plots for electrochemical impedance spectroscopy (EIS) results were also evaluated in the 0.01–100 kHz frequency span, and the outcomes are shown in Fig. 22(a). Half-circles and arcs and lines that are situated in high and low-frequency zones make up the Nyquist plot. Ion diffusion is illustrated by lines and is related to the Warburg resistance (*Z*_w_). The *Z*_w_ value decreases as the line slope increases.^155^ The NiBTC@GO hybrid supercapacitor electrode's specific capacitance is plotted against GO concentration in Fig. 22(b). The GO concentration is influenced by the Ni element's concentration. Compared to NiBTC, the slope of NiBTC@GO2 is noticeably steeper. Fig. 22(c) contains an illustration of the corresponding analog circuit. Owing to the simulated findings, the aggregate electrolytic solution resistance (*R*_s_) corresponding to NiBTC and NiBTC@GO2 are 0.6602 Ω and 0.7911 Ω, respectively. The diameter that defines the semicircle in the a high-frequency domain demonstrates the charge transfer resistance (*R*_ct_) resulting from the redox processes. The lowest semicircle diameter is found in NiBTC@GO2. The equivalent electrical conductivity values of NiBTC and NiBTC@GO2 are 0.131 and 0.142 S m^−1^, respectively, and their respective *R*_ct_ values are 7.649 Ω and 7.065 Ω. Interestingly, NiBTC@GO2 has good cycling performance; even after 5000 cycles, it maintains 84.47% of its initially generated capacity as opposed to 75.81% for NiBTC (Fig. 22(d)). In a nutshell NiBTC@GO2 indicates outstanding impedance properties, with the electrolyte's OH^−^ ions possessing a more rapid ability to diffuse to the electrode surface. GO can improve the composites' conductivity and offer sufficient OH^−^ embedding and dis-embedding space, whereas Ni-BTC can reduce GO aggregation and provide a uniformly stiff framework for GO adhesion.

## 7 Smart supercapacitor and artificial intelligence (AI) energy storage devices

Smart supercapacitors refer to advanced energy storage devices that go beyond the traditional capabilities of conventional supercapacitors. While energy storage remains a primary function, smart supercapacitors must possess additional functionalities and features, making them “smart” in the context of modern technological advancements that enable them to sense, adapt, or respond to changes in their environment or user requirements. Smart supercapacitors require the electrode and electrolyte materials to exhibit not just high conductivity and wide surface area, but also intelligent behaviors.^156^ MOF/GO composites benefit from the mechanical robustness of graphene oxide and exhibit stimuli-responsive behavior, such as reversible structural changes in response to external stimuli like temperature, pressure, or chemical exposure. MOFs can also serve as sensors due to their ability to selectively adsorb target molecules, and thus by combining MOF/GO composites with appropriate sensing elements, smart supercapacitors can detect specific analytes or environmental conditions while simultaneously storing energy. GO, well known for its flexibility, makes MOF/GO composites well-suited for integration into flexible and wearable electronics,^157^ and smart supercapacitors based on these composites can be conformably integrated into various form factors, enabling applications in Internet of Things (IoT) devices (*i.e.* Artificially Intelligent devices),^158,159^ renewable energy^160^ and beyond.^161–163^

**Fig. 23:**
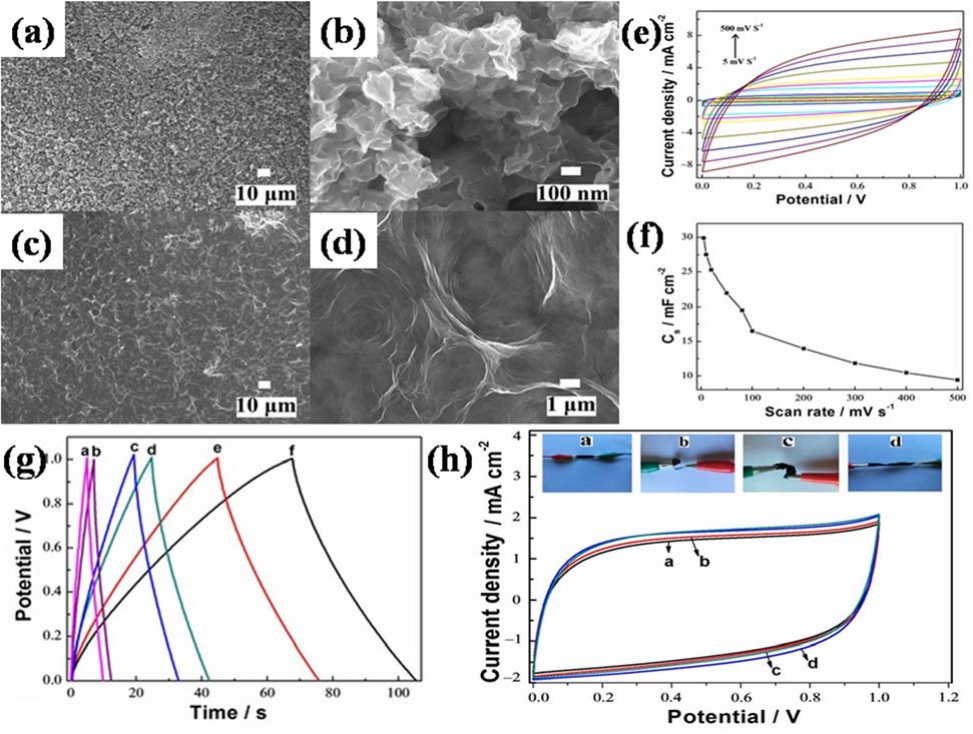
(a) Scanning electron microscopy images of (a) and (b) P@66C, (c) and (d) PGO@66C, (e) the PGO@66C solid-state SC device's cyclic voltammetry curves spanning from 5 mV s^−1^ to 500 mV s^−1^ at various scan speeds, (f) relationship between the PGO@66C solid-state SC device's areal capacitance and cyclic voltammetry scan rates, (g) the solid-state SC device PGO@66C's galvanostatic charge/discharge curves at various GCD current densities (a: 5, b: 2, c: 1, d: 0.8, e: 0.5, f: 0.4 mA cm^−2^), and (h) cyclic voltammetry curves obtained for the PGO@66C solid-state SC device under various curvature (a: flat; b, c: bent; and d: twisted) circumstances at a scan rate of 50 mV s^−1^.^164^ (a)–(h) Adapted with permission from source: *ChemistrySelect*, 2016, **1**, 285–289. Copyright 2016 Wiley Online Library.

Fu *et al.* synthesized a metal organic framework (UiO-66), as an illustration, based on zirconium and inscribed onto a carbon nanotube membrane (CNTF), which is referred to as 66-C.^164^ After that, PEDOT-GO or poly(3,4-ethylenedioxythiophene)-graphene oxide (PGO) was codeposited electrochemically on 66-C to obtain a porous electrode that was extensible (PGO@66C), which had excellent properties for flexible supercapacitors. There has never been a report on the composites that combine MOFs with PGO as electrode materials used in solid-state supercapacitor devices that offer flexibility. The morphology and microstructure of the developed samples were examined by SEM. Fig. 23(a) and (b) displays the P@66C SEM images. We can observe that, upon electrodeposition, conductive PEDOT covered and connected UiO-66, and the films display the porosity feature. The PGO@66C SEM micrographs in Fig. 23(c) and (d) reveal a considerable shift in morphology compared to Fig. 23(a) and (b). The interface of the sheets displays a curled bend that is compatible with the microstructure of GO when the composites undergo polymerization in the presence of GO. The degree of porosity of P@66C can be raised by the loose flexible microstructure resembling a sheet of PGO@66C, which would enhance electrochemical characteristics and facilitate electrolyte access. Fu's group fabricated a solid-state symmetrical SC device and employed two-electrode system to examine its functioning and efficiency. The CV curves of the PGO@66C device at various speeds of scans extending from 5 to 500 mV s^−1^ are illustrated in Fig. 23(e). It is evident that the PGO@66C device's CV curves exhibit quasi-rectangular profiles with a significant rise in current as scan speeds increase. The PGO@66C gadget exhibits an enormous rate capability, according to the results. Areal capacitance contrary to CV scan rates for the PGO@66C device is displayed in Fig. 23(f). As the scan rates increase, the areal capacitance values decline. At 5 mV s^−1^, the device's areal capacitance values reach 30 mF cm^−2^. Fig. 23(g) shows the device's triangular GCD curves with a current density between 0.4–5 mA cm^−2^. For all GCD current densities, the PGO@66C device shows a reduced *iR* drop. The solid-state capacitor device's strong capacitive qualities are demonstrated by the symmetry and linear contour of the charging and discharging curves. An energy density of 0.0022 mW h cm^−2^ and a power density of 0.2 mW cm^−2^ were attained at a current density of 0.4 mA cm^−2^. As depicted in Fig. 23(h), the solid-state capacitive component was evaluated at a scanning rate of 50 mV s^−1^ in a range of various bending and twisting situations. Because of its extreme flexibility and light weight, the SC may be bent and twisted in any way without losing its structural integrity. Additionally, the CV profile (Fig. 23(h)) showed the device's exceptional mechanical qualities as it stays constant and even gets larger at various bending angles. The characteristics of PGO@66C indicate that MOF-based solid-state capacitors hold great promise for applications in flexible and wearable AI electronics.

**Fig. 24:**
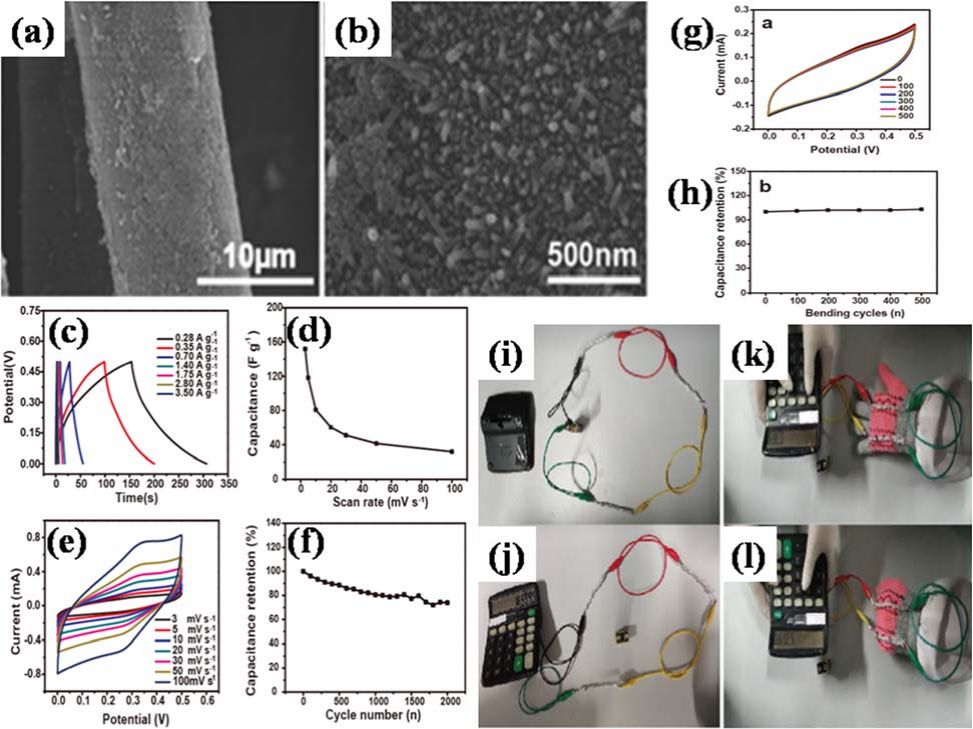
(a) and (b) Scanning electron microscopy micrographs of CuMOF/PET@rGO yarns, (c) galvanostatic charging/discharging curves of CuMOF/PET@rGO electrode, (d) specific capacitances of CuMOF/PET@rGO electrode, (e) cyclic voltammetry curves of CuMOF/PET@rGO electrode, (f) cycling stability of the CuMOF/PET@rGO electrode for 2000 cycles, (g) mechanical bending's impact on the CV curves, (h) effect of mechanical bending on capacitances, and pictures of an electronic calculator in the (i) and (j) fixed state, (k) and (l) bending state, with the assembled yarn SCs serving as the power source.^165^ (a)–(l) Adapted with permission from source: *Energy Fuels*, 2020, **34**, 16879–16884. Copyright 2020 American Chemical Society.

Yue *et al.* synthesized yarn electrode by cultivating conductive clusters of MOF nanorods covered in reduced graphene oxide-coated polyester (PET@rGO) yarn.^165^ They synthesized GO sheets using modified Hummers' method and then immersed the cleaned PET yarn in a well-dispersed GO solution to create an incredibly thin GO layer over its surface. Thereafter, it was dried in a vacuum at 50 °C for 3 hours and this procedure of dipping sheets and subsequently drying them was replicated for ten cycles. In an ascorbic acid solution, the GO film was chemically decomposed at 90 °C while being stirred. The resultant PET@rGO yarn was rinsed after four hours by vacuum-drying it at 50 °C and washing it three times in DI water. Then they synthesized CuMOF/PET@rGO yarn electrodes by adding Cu(C_2_H_3_O_2_)_2_·H_2_O and HHTP in DMF/DI water mixture in certain ratios. In the above solution, the PET@rGO yarn was introduced. After undergoing a 12 hour reaction at 85 °C, the CuMOF/PET@rGO yarn was carefully washed three times with deionized water and allowed to naturally dry at room temperature. A 3D nanostructured array layer made up of many CuMOF nanorods is visible on the PET@rGO fiber surface in Fig. 24(a) and (b). The average diameter of these nanorods is 20 nm. Large active surfaces can be exposed more easily because of this special shape, which also creates an effective diffusion path for electrolyte ions. At varying scanning rates, the CV curves have been described in Fig. 24(e). Each curve exhibited two mild anodic and cathodic peaks, suggesting that Cu^2+^/Cu^+^'s pseudocapacitive behavior is a component of the electrochemical mechanism. The symmetric redox peaks demonstrate the high reversibility of the CuMOF/PET@rGO electrode. Even at high scan rates, the CV curve morphologies remained similar, suggesting strong rate performance. When the scan speeds were raised, there was a possible shift in the anodic and cathodic peaks, which is connected to the electrode polarization. The GCD curves acquired at different current densities are displayed in Fig. 24(c). Their almost triangular shape suggests strong electrochemical activity and the pseudocapacitive contribution could be the cause of the GCD curves' divergence from linearity. The determined specific capacitances are plotted in Fig. 24(d) based on the outcome of the CV measurement. With the CuMOF/PET@rGO electrode's superior structural characteristics providing assistance, a specific capacitance of 151.9 F g^−1^ at 3 mV s^−1^ was established. To assess the cycling stability of the CuMOF/PET@rGO electrode, it was continuously charged and discharged (Fig. 24(f)). Despite a noticeable decrease in capacitance performance due to structural fatigue, the CuMOF/PET@rGO electrode maintained 74% of its initial capacitance following 2000 cycles. For 500 cycles, the CuMOF/PET@rGO electrode was bent regularly, and as can be seen in Fig. 24(g), there was an overlap in the recorded CV curves. Retentions of capacitance above 92% were computed (Fig. 24(h)), resulting from which the assembled yarn SC was used to supply electricity for small electrical gadgets, as well as an electronic calculator could be powered by two yarn SCs connected in series to carry out a mathematical calculation (Fig. 24(i) and (j)). Further fixing of these yarn SCs was done on a wool glove (Fig. 24(k) and (l)) and they were also able to activate the electronic calculator after bending it numerous times. Based on these findings, our yarn SCs appear to be a potential energy storage solution for wearable and flexible electronics.

### 7.1 IoT energy storage devices

Smart electronic artificial intelligence (AI) devices with a range of functions,^166–169^ including shape memory, electrochromism, photodetection, sensing capability, adaptability and self-monitoring, self-diagnostic and health monitoring, energy harvesting and storage, safety and fault tolerance, and user interface and human–machine interaction, have been developed recently.^170,171^ By incorporating responsive MOFs into the composite, smart supercapacitors can be designed to adapt their performance or trigger specific functionalities in response to environmental changes. Graphene oxide, despite being an insulator, can be functionalized or reduced to graphene to improve its electrical conductivity. Furthermore, graphene is more favorable for storing the charge than other carbon materials because of its enormous specific surface area (2630 m^2^ g^−1^), which remains constant regardless of the pore size variation.^172,173^ Incorporating graphene oxide into MOF-based composites helps create a conductive network throughout the material, facilitating rapid electron transfer and improving the overall performance of the supercapacitor.

Very recently, in the treatment of colorectal cancer, Shen *et al.* introduced a pH-responsive Fe-MOF@GO composite drug delivery system characterized by a stable structure and uniform particle size distribution.^174^ The incorporation of GO and MOF, both possessing lamellar structures, enhances drug retention and permeability, thereby promoting drug absorption. This study effectively demonstrates the potential of MOF@GO drug delivery systems in healthcare, including drug loading and anti-tumor applications. Furthermore, there were challenges in using 2D-MOFs for electrode preparation because of the strong van der Waals attraction of neighbouring nanosheets, which may cause the massive 2D-MOF films to aggregate and self-restack next to each other. This may seriously compromise their ease of access to electrolyte ions and could negatively impact devices' electrochemical performance.^175^ By carefully incorporating strongly conductive graphene layers into 2D-MOF films to create a switching graphene/MOF multilayer arrangement, Cheng's group suggested a method to improve the characteristics of 2D-MOF electrode materials.^176^ They showed how to develop hybrid electrodes using the electrostatic self-assembly method, which included self-assembled 2D Co-MOF/rGO paper. They also suggested a one-to-two approach, which involves employing a synthesis process developed from MOF to generate two different kinds of electrodes with good porosity for FSSCs: CoMOF@rGO and NiMOF@rGO. This process merely requires the metal ion precursors to be altered. An extensive asymmetric all-solid-state supercapacitor was created utilizing a negative NiMOF@rGO40 electrode, a positive CoMOF@rGO40 electrode, and an electrolyte made up of PVA-H_2_SO_4_, in order to create customizable supercapacitors possessing a large energy density. A remarkable areal capacitance of 426.5 mF cm^−2^ and a volumetric capacitance of 22.45 F cm^−3^ at 1.2 mA cm^−2^ were displayed by the constructed paper-based CM/rGO40//NM/rGO40 E-SCs. GCD experiments under various bending angles were performed to evaluate the device's flexibility. As compared to the original state, the specific capacitance could retain 97.5% during the procedure. Specifically, the specific capacitance remained at 93.9% regardless of 100 twisting rounds at a 180° bending angle which can be ascribed to the interconnections formed by the CM/rGO40//NM/rGO40 E-SC. The flexible gadget, measuring 35 mm by 20 mm, is remarkably thin at just 1.9 mm in thickness and so light that even a delicate flower stamen may lift it up. Notably, modifying the E-SCs device's design allowed for a demonstration of its modifiable performance, wherein the E-SCs could continue to light the LED even after being cut multiple times. Thus, the above-described results amply show that the paper-based CM/rGO40//NM/rGO40 E-SCs, which are all-solid-state, may function as a dependable and customizable energy storage device for wearable computer applications.

Overall, MOF/GO composites offer a versatile platform for the development of smart supercapacitors with enhanced performance, responsiveness, and versatility, opening up exciting opportunities for advanced energy storage applications. This combination exploits the excellent electrical conductivity and mechanical strength of GO along with the enormous surface area and tunable pore size of MOFs, resulting in a unique morphology with synergistic effects. The hybrid material enhances charge storage capacity, accelerates electron transport, and ensures structural stability, making it ideal for responsive and efficient smart supercapacitors. Ongoing research focuses on optimizing scalability and cost-effectiveness for practical applications in modern AI energy storage systems. This integration could open exciting possibilities for the evolution of smart supercapacitors with improved performance.

## 8 Conclusion and outlook

In this review, we gave a thorough rundown of the most recent advancements in MOF/GO-based electrode materials tailored for advanced energy storage applications. The discussion encompassed diverse characteristics, ranging from chemical synthesis methods to magnetic and non-magnetic metal-based MOFs, and various composites like MOF/G, MOF/GO, MOF/rGO. Emphasis was placed on their properties in the context of advanced energy storage applications of smart supercapacitors, shedding light on how these materials can propel progress in developing energy storage solutions with heightened performance and capabilities. To overcome the limitation posed by the low conductivity of organic linkers in MOFs, graphene and its derivatives are strategically combined with MOFs, enabling the enhancement of electrochemical properties through improved conductivity.

Various types of MOF/GO composites have been discussed herein, including Mo-MOF/GO, Fe_2_O_3_-MOF/GO, Ni/Co-MOF-rGO, ZIF-8/GO, Ce-MOF/GO, HMRL-1/rGO, and others. These composites are of great interest to researchers due to their exceptional electrochemical performance and other properties resulting from the synergistic interaction between MOF and GO architectures. Conversely, various composites of GO with other materials have been outlined, such as GO/ZHS, Chitosan/GO hydrogel, graphene quantum dots (GQDs), rGO/MXene-PPy, and others, that have also demonstrated promising potential in the realm of energy storage devices. Furthermore, discussions have extended to composites of MOFs with other materials, including trimetallic MOF-CNT, Co-MOF/PANI, MOF-PEDOT, MOF-LaFeO_3_, Cu-MOF@δ-MnO_2_, among others.

This article covers the types and operation of superconductors, rationale and properties of MOF/GO composites, their surface engineering, three-electrode, and two-electrode electrochemical characteristics, and their applications in smart supercapacitors. Selecting appropriate metal cations and linkers aids in fine-tuning the final structural characteristics for improved ion migration, as the covalent connection between MOF and GO is facilitated by a number of linkers. Rather than using the post-synthesis technique, hydrothermal or solvothermal synthesis, co-precipitation method and mixing method routes are typically used to guarantee a stronger contact between two materials, offering a better control over the composite's properties.

Lastly, GO-based nanostructures have demonstrated their distinctive qualities in MOF/GO composites for smart supercapacitors, according to data that have been presented, which represent a significant advancement beyond traditional energy storage devices, with additional functionalities enabling them to sense, adapt, and respond to changes in their environment or user requirements. Numerous MOF/GO composites, including PGO@66C, Fe-MOF@GO, MOF/PET@rGO yarn, 2D Co-MOF/rGO paper, among others, have been explored which exhibit promising stimuli-responsive properties and sensing capabilities, rendering them well-suited for smart supercapacitor development. Their potential integration into flexible and wearable electronics presents opportunities for diverse applications in IoT and renewable energy sectors.

The metal coordination environments of various MOF types vary greatly, which may have a substantial impact on how well they interact with GO layers. Determining the primary mechanism in the synthesis of a certain MOF/GO compound remains difficult to this day as all MOF/GO composite materials, however, might very well have their own distinct MOF crystallization mechanism and production pathway. Therefore, it is highly recommended to conduct additional fundamental study in order to solidify existing knowledge in this area and, more crucially, investigate any gaps or new information on GO-guided MOF growth. The main issue with GO nanosheets is their restacking, which restricts their applicability by keeping a significant section of their surfaces unavailable for charge storage and electrolyte diffusion. Future research should concentrate on creating mechanically stable and flexible electrodes for use in small, flexible IoT energy storage devices. Even though the synthesis of MOF/GO composites has advanced significantly, in-depth examinations are still required to fully assess their intriguing architectures. Proportionately, we discovered that more study in the following fields would be quite beneficial because it has always been desirable to conduct additional research on the uses of dedicated MOF/GO composites, with the knowledge of controlling the MOF structure and orientation, which are anticipated to exhibit advanced characteristics in smart supercapacitor applications. Overall, this review attempted to address the fundamental knowledge of the underlying MOF/GO composite formation mechanisms and the overall goal of this review was to address basic understanding of the mechanics behind the creation of these composites, wherein we discovered that the study on MOF/GO composites is still in stages of development and that there are surprisingly few studies in this developing sector. Given the field's quick progress, this particular category of MOF/GO composite materials has the potential to evolve into an increasingly popular subject among academics which could open up numerous additional intriguing applications in the years to come.

## Author contributions

S. R. did methodology, investigation, discussions and writing – original draft & editing, and S. G. did conceptualization, investigation, formal analysis, writing – review & editing, funding acquisition, project administration and supervision, N. G. and S. P. did investigation, and discussion.

## Conflicts of interest

There are no conflicts to declare.

## Supplementary Material
